# Salvia Miltiorrhiza Root Water-Extract (Danshen) Has No Beneficial Effect on Cardiovascular Risk Factors. A Randomized Double-Blind Cross-Over Trial

**DOI:** 10.1371/journal.pone.0128695

**Published:** 2015-07-20

**Authors:** Pleun C. M. van Poppel, Pauline Breedveld, Evertine J. Abbink, Hennie Roelofs, Waander van Heerde, Paul Smits, Wenzhi Lin, Aaitje H. Tan, Frans G. Russel, Rogier Donders, Cees J. Tack, Gerard A. Rongen

**Affiliations:** 1 Department of Internal Medicine, Radboud university medical center, Nijmegen, The Netherlands; 2 Clinical Research Centre Nijmegen, Radboud university medical center, Nijmegen, The Netherlands; 3 Department of Gastro-Enterology, Radboud university medical center, Nijmegen, The Netherlands; 4 Department of Clinical Chemistry, Radboud university medical center, Nijmegen, The Netherlands; 5 Department of Pharmacology and Toxicology, Radboud university medical center, Nijmegen, The Netherlands; 6 Department for Health Evidence, Radboud university medical center, Nijmegen, The Netherlands; 7 Medical Center Balans, The Hague, The Netherlands; 8 Practice for acupuncture and member of the Dutch Association of Acupuncture Medicine, Amsterdam, The Netherlands; University of Leicester, UNITED KINGDOM

## Abstract

**Purpose:**

Danshen is the dried root extract of the plant Salvia Miltiorrhiza and it is used as traditional Chinese medicinal herbal product to prevent and treat atherosclerosis. However, its efficacy has not been thoroughly investigated. This study evaluates the effect of Danshen on hyperlipidemia and hypertension, two well known risk factors for the development of atherosclerosis.

**Methods:**

This was a randomized, placebo-controlled, double-blind crossover study performed at a tertiary referral center. Participants were recruited by newspaper advertisement and randomized to treatment with Danshen (water-extract of the Salvia Miltiorrhiza root) or placebo for 4 consecutive weeks. There was a wash out period of 4 weeks. Of the 20 analysed participants, 11 received placebo first. Inclusion criteria were: age 40-70 years, hyperlipidemia and hypertension. At the end of each treatment period, plasma lipids were determined (primary outcome), 24 hours ambulant blood pressure measurement (ABPM) was performed, and vasodilator endothelial function was assessed in the forearm.

**Results:**

LDL cholesterol levels were 3.82±0.14 mmol/l after Danshen and 3.52±0.16 mmol/l after placebo treatment (mean±SE; p<0.05 for treatment effect corrected for baseline). Danshen treatment had no effect on blood pressure (ABPM 138/84 after Danshen and 136/87 after placebo treatment). These results were further substantiated by the observation that Danshen had neither an effect on endothelial function nor on markers of inflammation, oxidative stress, glucose metabolism, hemostasis and blood viscosity.

**Conclusion:**

Four weeks of treatment with Danshen (water-extract) slightly increased LDL-cholesterol without affecting a wide variety of other risk markers. These observations do not support the use of Danshen to prevent or treat atherosclerosis.

**Trial Registration:**

ClinicalTrials.gov NCT01563770

## Introduction

Worldwide, cardiovascular disease (atherosclerosis) is the leading cause of mortality[1]. Traditional risk factors for atherosclerosis are hyperlipidemia, hypertension, diabetes mellitus, smoking and inflammation, which has gained much attention more recently[2, 3]. In both primary and secondary prevention, treatment of hypertension and hyperlipidemia reduces the risk of cardiovascular events[4–6].

Traditional Chinese Medicine (TCM) has been playing an important role in healthcare in Asia for hundreds of years. Recently, there is an upsurge in the popularity of TCM products among consumers outside China. In 2010, the United States spent $7.6 billion on TCM products and Europe $2 billion [7]. Factors that contribute to the increased use of TCM products are the increased prevalence of obesity and related chronic disorders, increasing costs of conventional medicines and the belief that TCM products are safer and more effective than prescription drugs [8].

Danshen is the dried root extract of the plant Salvia Miltiorrhiza and is widely used in Asia to treat coronary artery disease, hyperlipidemia and cerebrovascular disease[9]. The estimated number of patients using Danshen is approximately 5 million worldwide[10]. According to TCM theory, Danshen is used in patients to treat “blood stasis” as it “moves blood” [11]. The TCM-qualification of “blood stasis” is made by identification of certain changes in the tongue and pulse. The major components of Danshen are hydrophilic salvianolic acids and lipophilic tanshinones[12]. In vitro treatment of human monocyte derived macrophages with a combination of Danshen and Gegen resulted in a suppression of acetylated Low Density Lipoprotein (LDL) uptake [13]. Two active components of Danshen, salvianolic acid A and magnesium tanshinoate B, inhibit LDL oxidation [11]. A mixture of Chinese herbs, including Danshen, has been shown to exert beneficial effects on high-fat diet induced metabolic syndrome in rats [14]. Also in humans, Danshen may reduce LDL-levels and blood pressure [11]. However, the quality of randomized clinical trials of Danshen is poor [15–17] and not easily accessible to Western physicians since most are published in Chinese.

Therefore, this study was designed to evaluate the effect of Danshen on hyperlipidemia and hypertension, two well validated risk factors for cardiovascular disease according to evidence-based medicine.

## Patients and Methods

### Study Population

**Fig 1:**
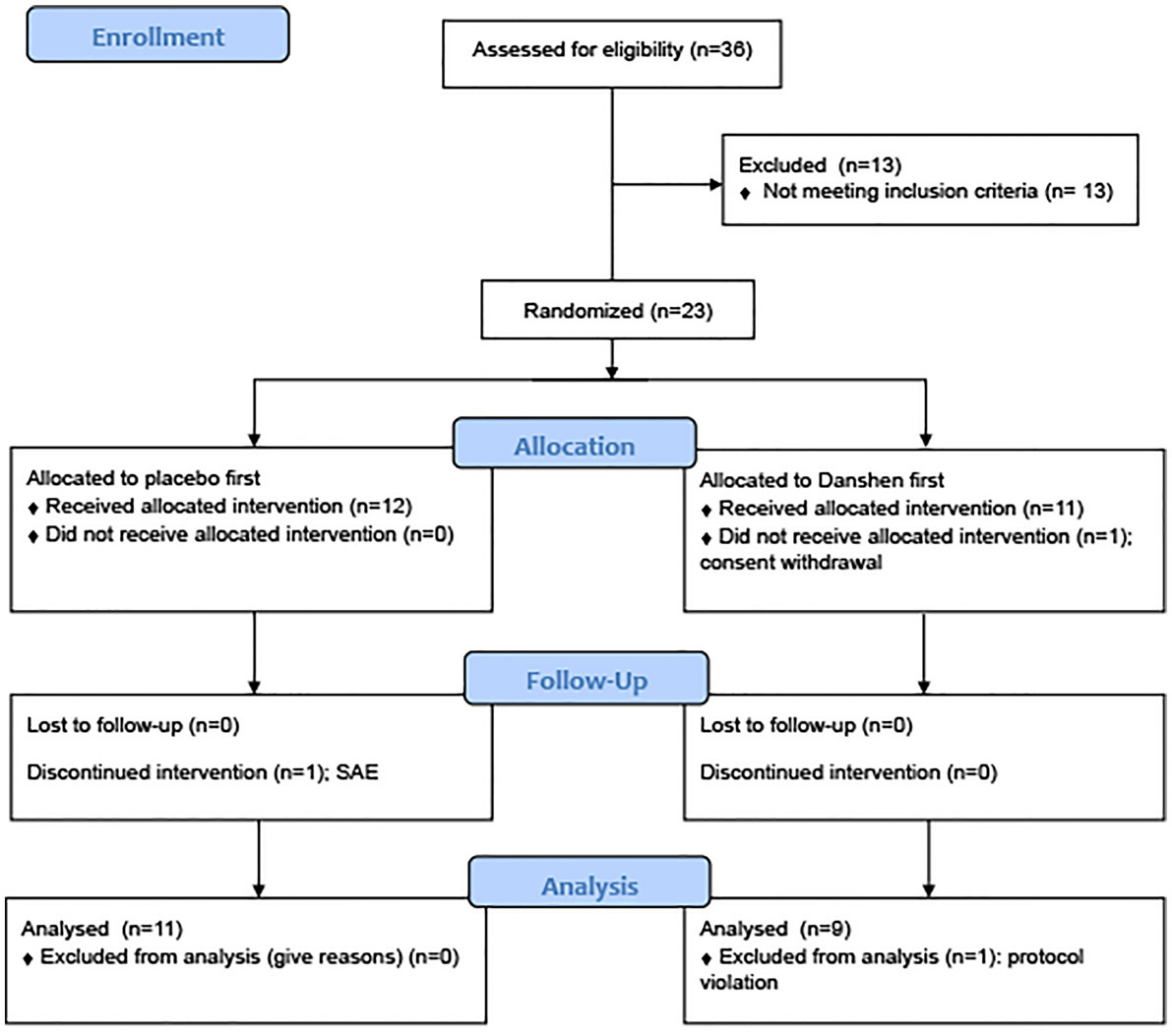
CONSORT flow diagram. SAE: Serious Adverse Event. The SAE (Bell’s palsy) occurred during Danshen treatment (see text).

The study population consisted of 20 subjects aged 40–70 years with hyperlipidemia and hypertension, who were recruited through advertisements in local newspapers (Fig 1). Included were subjects with a fasting LDL cholesterol > 3.5 mmol/l and/or triglycerides > 1.7 mmol/l. Patients treated with antihypertensive drugs were eligible when the average of three office blood pressure recordings (sphygmomanometer, sitting position, at least 5 minutes interval) revealed a systolic blood pressure > 140 mmHg and/or diastolic blood pressure > 90 mmHg. For subjects that were not treated with antihypertensives, blood pressure was recorded three times on two separate visits (in total 6 recordings, in sitting position, sphygmomanometer, 5 minute intervals between recordings on each visit) and again average blood pressure had to fulfill the criterium of > 140 mmHg for systolic and/or > 90 mmHg for diastolic blood pressure. Excluded were subjects with triglycerides > 8 mmol/l and/or LDL-cholesterol > 5 mmol/l or systolic blood pressure > 180 mmHg and/or diastolic blood pressure > 110 mmHg. Further exclusion criteria were treatment with statins, use of more than 1 antihypertensive drug, use of angiotensin-converting-enzyme inhibitors, angiotensin II receptor antagonists, calcium antagonists or vitamin and/or herbal supplements, diabetes mellitus treated with insulin, a history of cardiovascular disease, use of anticoagulant drugs, a serum alanine aminotransferase or aspartate aminotransferase level of more than 3 times the upper limit of normal and abnormal creatinin clearance defined as MDRD< 60 ml/min/1.73m^2^. The exclusion of angiotensin converting enzyme inhibitors, angiotensin II receptor antagonists and calcium antagonists was based on documented improvement by these drugs of acetylcholine-induced forearm vasodilation, as opposed to non-dilating beta blockers and thiazide diuretics [18–21]. Therefore, failure to exclude these drugs could bias acetylcholine-induced vasodilation which was one of the secondary endpoints.

### Protocol

**Fig 2:**
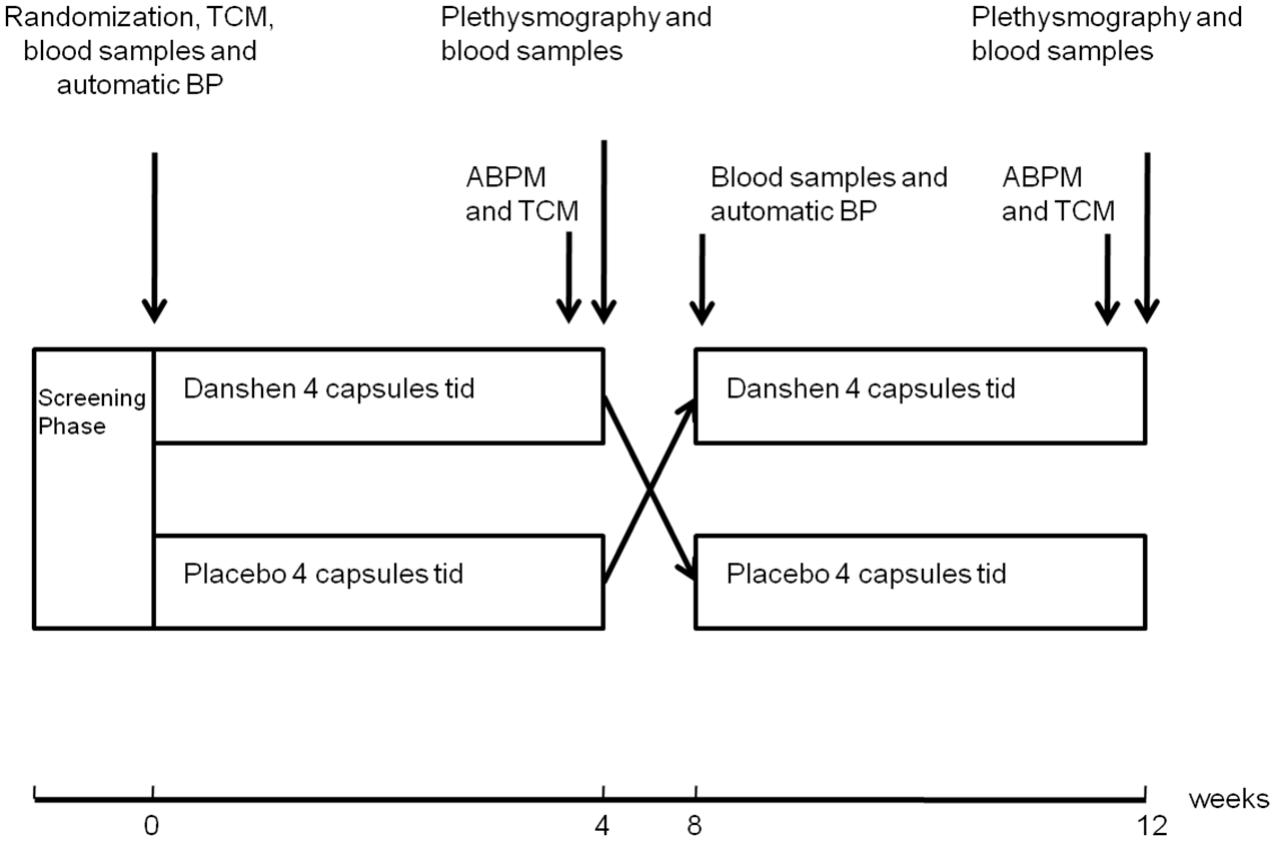
Graphic presentation of the study protocol. Four weeks treatment with Danshen and placebo in a crossover design with at least four weeks of washout (represented in the figure as the period between week 4 and week 8). BP = blood pressure. ABPM = 24-h Ambulatory Blood Pressure Monitoring. TCM = traditional Chinese Medicine diagnosis.

This was a randomized, double-blind, placebo controlled crossover study performed at a tertiary referral center (Fig 2). After inclusion in the study, subjects were randomly assigned to treatment with Danshen capsules, 4 capsules of 500 mg tid, for 28 days or placebo capsules, 4 capsules tid, for 28 days. After a washout period of 4 weeks participants crossed over to the other treatment arm. Investigational medication was given on top of their other medication.

Participants were contacted by telephone halfway each treatment period to check for compliance and possible side effects.

At the end of each treatment period blood was collected, blood pressure, body weight and endothelium-mediated vasodilation were measured, and potential side effects were recorded. Compliance was monitored by pill counts. Prescribed medication was not altered during the trial and participants did not change their dietary habits. Plasma lipids were the primary outcome and blood pressure was the secondary outcome of this trial. All other outcome parameters are exploratory.

### *Salvia miltiorrhiza* root water-extract (Danshen), randomization procedure and blinding

*Salvia miltiorrhiza* extract was produced by Kaiser Pharmaceuticals Co. in Taiwan. This product was obtained from a specialized pharmacy NatuurApotheek. Using this extract, Basic Pharma Manufacturing BV produced Danshen and placebo capsules. Each capsule contained 500 mg granulate consisting of 50% water-extract of Danshen and 50% starch. Therefore, a dosage of 4 capsules tid with 500 mg granulate is equal to 4 capsules with 250 mg extract tid (3 g daily). Since the root—extract ratio is 5:1, 3 gram extract originates from 15 g root. The Pharmacopoeia of the People’s Republic of China recommends a dosage of 9–15 g root daily [10]. So, we used in this study the highest dose of Danshen that is regularly used in TCM. In this way, we wanted to reduce any doubts about insufficient dosing in case of lack of efficacy or overdosing in case of unexpected adverse events.

Briefly, the production process of Danshen is as follows: first a water decoction of Salvia root is made. Subsequently, the water-extract is concentrated 5 times via a standard distillation procedure. After the concentration procedure, a drying and granulation procedure (with 50% starch as carrier) followed using a fluid bed spray dryer. Then the granules are filtered, which results in a final bulk product consisting of granules of a specified size. Finally, the bulk product is filled into containers. The NatuurApotheek also prepared the placebo’s according to Good Manufacuring Practice and filled these in equally appearing containers. Placebo and Danshen capsules were similar in appearance. The labels of the containers included the randomization number (two containers (placebo and Danshen) per randomization number), and period number. The Natuurapotheek performed the randomization. The randomization list which linked randomization number to treatment order, was stored at a safe place at the Clinical Research Centre Nijmegen. Participants received a number according to inclusion order and received the medication with randomization number that equaled the inclusion number. The research team involved in recruitment, assessment of primary, secondary and explorative outcome measurements (including laboratory personnel) and data entry as well as participants remained blinded until all data had been entered in the database and the database had been monitored by an independent trial monitor.

### Biochemical analyses

#### Lipids

Blood was drawn in fasting state before and after each treatment period to measure total cholesterol, triglycerides, HDL-cholesterol and apolipoprotein B. The LDL- cholesterol was calculated using the Friedewald equation (LDL-cholesterol = total cholesterol—HDL-cholesterol– 0.45 x triglycerides) [22]. Total cholesterol and triglyceride concentrations were measured using an enzymatic method. For determination of HDL-levels, direct immunoinhibition was used. Apolipoprotein B levels were determined by turbidimetry (all Architect, Abbott Diagnostics Division). Coefficients of variation were for total cholesterol <3%, triglycerides <5%, HDL-cholesterol ≤4% and for apolipoprotein B ≤6.5%.

#### Metabolic and inflammatory parameters

Glucose concentrations were measured using the glucose oxidase-peroxidase method. (Architect, Abbott Diagnostics Division). Insulin levels were determined by an electrochemiluminescence immunoassay (Modular E170, Roche Diagnostics). The coefficients of variation were ≤ 5% for glucose and 3.2% for insulin.

Homeostasis model assessment of insulin sensitivity (HOMA IR) was calculated using the following formula: fasting glucose (mmol/l) x fasting insulin (mU/l)/ 22.5. For Homeostasis Model Assessment (HOMA) of beta cell function (HOMA-B) the formula was: 20x fasting insulin (mU/l)/ fasting glucose (mmol/l)-3.5 [23].

As marker of oxidative stress, lipid peroxidation was determined by measuring ThioBarbituric Acid Reactive Substances (TBARS) in plasma using a fluorimetric assay [24]. Furthermore, antioxidant capacity was determined by means of Ferric Reducing Ability of Plasma assay (FRAP) [25].

Measurements of safety parameters (hemogram, electrolytes, creatinin and liver enzymes) were performed at the department of Laboratory Medicine, Radboud university medical center.

### Blood pressure measurement

Before start of study medication, blood pressure was measured for 20 minutes at two minute intervals by an automated device (Nihon Kohden BSM 2301, Nihon Kohden, Japan) using the oscillometric principle with the subject in supine position.

Furthermore, in the last week of each treatment period blood pressure was measured at home by 24-h Ambulatory Blood Pressure Monitor (ABPM) using a fully automated device (Mobil-O-Graph NG, I.E.M. GmbH, Germany). ABPM results were expressed as mean total, day (from 8.00 a.m. to 10.59 p.m.) and night (from 11.00 pm to 7.59 a.m.) blood pressure.

### Forearm vasodilator response to acetylcholine and sodium nitroprusside

Forearm blood flow (FBF) was assessed by venous occlusion plethysmography using mercury-in-silastic strain gauges (Hokanson EC4. Hokanson, Inc) as previously described [26]. The experiments started at 8.30 a.m. after an overnight fast in a quiet, temperature controlled room (23°C–24°C). Study medication was ingested at home, prior to visiting our clinical research department. The subjects were studied after 24 hours of caffeine abstinence. The brachial artery of the non-dominant arm (experimental forearm) was cannulated (27-gauge needle, Braun, Melsungen, Germany) for infusion of vasodilators. FBF was measured at both forearms simultaneously. The upper arm cuffs were inflated using a rapid cuff inflator (Hokanson E-20, DE Hokanson, Bellevue, WA). Wrist cuffs were inflated to 220 mm Hg to occlude the hand circulation during the infusion of both vasodilators and their baselines [27]. Forearm volume was measured with the water displacement method and all drugs were dosed per 100 ml forearm tissue with a constant infusion rate of 100 μl.dl^-1^.min^-1^ during infusion of solvent (baseline recordings) as well as during infusion of increasing doses of vasodilators

After complete instrumentation, a 30 minute equilibration period followed, after which baseline measurements were performed with infusion of saline. Subsequently, three increasing doses of acetylcholine (0.5, 2.0 and 8.0 μg.dl^-1^.min^-1^, 10 mg/ml dry powder, dissolved to its final concentration with saline, Novartis, Greece) were infused into the brachial artery. Acetylcholine stimulates endothelial muscarinic receptors thereby activating nitric oxide synthase. This results in endothelial release of nitric oxide (NO) causing vasodilation[26]. Each dose was infused for 5 minutes and FBF was measured. After the last acetylcholine dose, a 30 minutes equilibration period followed. Subsequently, baseline measurements were performed with the infusion of glucose 5% solution. Subsequently, three increasing doses of sodium nitroprusside (0.06, 0.20 and 0.60 μg.dl^-1^.min^-1^, 25 mg/ml, dissolved to its final concentration with glucose 5% solution, Clinical Pharmacy, Radboud university medical centre), a NO-donor and endothelium-independent vasodilator [28] were infused. Again, each dose was infused for 5 minutes and FBF was measured. FBF registrations of the last 2 minutes of each dosage of vasodilator were averaged to a single value for data analysis.

### Hemostatic and rheological parameters

Screening for primary hemostasis abnormalities was performed using the platelet function analyzer (PFA-100, Siemens, Germany). The closure time was measured with the collagen epinephrine and collagen-ADP cassette. Normal reference ranges for the collagen epinephrine cassette is ≤ 170 sec with an inter assay variation of 12.4%. Normal reference ranges for the collagen ADP cassette is ≤ 120 sec with an inter-assay variation of 12.7%.

Von Willebrand Factor (vWF) antigen levels were measured using the Asserachrom VWF:Ag ELISA from STAGO. The Lower Limit Of Quantification (LLOQ) of this assay is 12.5% with an inter-assay variation coefficient of 4.7%. Reference intervals are blood group dependent and ranges from 50–150% (blood group O) up to 70–210% (blood group AB).

The effect of Danshen on coagulation and fibrinolysis was measured using the Nijmegen Hemostasis Assay which measures thrombin and plasmin generation in time [29]. The thrombin generation curve represents the following parameters: (i) lag-time, the time to the start of thrombin formation; (ii) thrombin peak time, i.e. the time when thrombin production reaches maximal velocity; (iii) thrombin peak height, the maximal velocity of thrombin generation; and (iv) the area under the curve (AUC) for the total amount of thrombin formed. Plasmin generation is described by: (v) fibrin lysis time, the time between the initiation of thrombin generation and the time plasmin generation reaches maximal velocity; (vi) plasmin peak-height, the maximal velocity of plasmin production; and (vii) plasmin potential, area under the curve that represents the total amount of plasmin generated. The inter-assay variation of thrombin generation parameters varies from 5.9% (AUC) to 25% (lag time thrombin generation). The inter-assay variation of plasmin generation parameters varies from 10% (plasmin peak height) to 14% (plasmin potential).

All hemostatic parameters were measured at the Department of Laboratory Medicine of the Radboud university medical Centre.

Blood viscosity was determined in vitro at a temperature of 37°C and at a shear rate of 3.16 s^-1^ with the viscometer LS300 (ProRheo GmbH, Germany).

### TCM-qualification of “blood stasis”

Two TCM practitioners independently determined the presence of “blood stasis” by pulse and tongue diagnosis and interrogation at baseline and after both treatment periods. The TCM practitioners were asked to categorize patients as having definite “blood stasis” (score 1), definite absence of “blood stasis” (score 3) or some characteristics of “blood stasis” but not fulfilling the criteria for a full qualification (score 2). Kappa values and polychoric correlations were calculated to estimate the agreement of both TCM-practitioners for the three visits separately. The polychoric correlation was calculated in addition to kappa-value since the distribution of the diagnostic score appeared not homogeneous with overpresentation of score 2, a situation that makes the kappa-value very sensitive to a single disagreement.

### Calculations and statistical analysis

We considered a change of 0.225 mmol/l in primary endpoint LDL-cholesterol between treatment groups as clinically relevant. Assuming a test-to-test correlation coefficient of 0.7 we would require a total of 20 subjects to detect a change of 0.225 mmol/l in LDL-cholesterol with a power of 80% at a significance level of 0.05 (Zα = 1.96). When the test-to-test correlation was 0.5, a study including 20 participants would be able to detect a difference of 0.3 mmol/l. Drop-outs were replaced.

Differences in means of laboratory results and blood pressure measurements were tested by paired Student’s t-test or Wilcoxon signed rank test for non-normally distributed data. In addition, the effect of Danshen on plasma lipids and ApoB-lipoprotein was analyzed with the CROS analysis according to Senn [30]. For each treatment period, we calculated the difference between before- and after measurement. Next, the difference between period one and period two was calculated. These differences were compared between the two randomisation groups using an independent sample t-test. To explore potential carry-over effects, we investigated treatment by period interactions. For this purpose, we compared the placebo-effect between the two randomisation groups as well as the Danshen-effect with an independent samples t-test.

Repeated measures analysis of variances (ANOVA) was used to assess the effect of Danshen on FBF-response to acetylcholine and nitroprusside. Statistical analyses were performed using Graphpad 5.0. Results are expressed as mean ± Standard Error of the Mean (SEM), unless otherwise indicated. Significance was set at a two-sided p-value of less than 0.05.

### Ethics statement

This study was conducted in accordance with the principles outlined in the Declaration of Helsinki and Good Clinical Practice (GCP) guidelines. The local Institutional Review Board (‘Commissie Mensgebonden Onderzoek’(CMO) Arnhem/Nijmegen, registration number: CMO 2011/326) approved the study and all subjects gave written informed consent before participation. The final protocol including the final amendments as marked in tracked changes is provided as supplemental information. The trial was registered at clinicaltrials.gov (NCT01563770).

## Results

The first participant was included on May 10, 2012. The 20^th^ fully evaluable and last participant visited our research centre on March 28, 2013. Twenty-three of the 36 initially screened subjects underwent randomization and were enrolled in the study. One participant withdrew before start of the study medication. One participant discontinued the intervention due to a facial nerve paralysis during Danshen treatment. Furthermore, one subject was excluded from statistical analyses because of insufficient compliance to the trial protocol. See Table 1 for baseline characteristics of the remaining 20 evaluable participants. All 20 evaluable participants were included in all analyses.

**Table 1:** Baseline characteristics.

Characteristics of 20 participating subjects (mean ± SD)	
Age (years)	58.0±7.7
Sex (male:female)	14:6
Weight (kg)	86.4±18.9
BMI (kg/m^2)^	28.6±5.6
Blood pressure systolic (mmHg)	156±9
Blood pressure diastolic (mmHg)	94±5
Total cholesterol (mmol/l)	6.2±0.7
Triglycerides (mmol/l)	1.9±1.3
HDL- cholesterol (mmol/l)	1.3±0.4
LDL- cholesterol (mmol/l)	4.0±0.5
Apolipoprotein B (g/l)	1.1±0.2
Fasting glucose (mmol/l)	5.5±0.6
HbA1c (%)	5.7±0.3
Antihypertensives (%)	n = 10 (50) Diuretics n = 6 (30), Beta-blockers n = 4 (20)

### Lipids

Danshen treatment did not affect High Density Lipoprotein (HDL) cholesterol, triglycerides and Apolipoprotein B. However, total cholesterol and LDL cholesterol were significantly higher after Danshen treatment compared to placebo, approximately 0.3 mmol/l (Table 2). Baseline levels of total cholesterol and LDL cholesterol were not different for both treatment periods. Furthermore, total cholesterol and LDL cholesterol were slightly but significantly higher after treatment with Danshen compared to baseline values (p < 0.01) while placebo treatment did not change baseline levels. When the effect on Danshen was expressed as change from baseline and corrected for the changes observed during placebo treatment, LDL-cholesterol was significantly increased (Table 3) while other lipid fractions were not affected. Analysis of potential carry-over effects did not reveal any significant treatment by period interactions.

**Table 2:** Lipid levels before and after treatment with danshen and placebo. Results are provided as mean±SEM. Baseline values did not significantly differ between Danshen and placebo treatment.

	Danshen		Placebo	
	Baseline	Treatment	Baseline	Treatment
Total cholesterol (mmol/l)	5.61±0.15	5.84±0.18* ^#^	5.60±0.19	5.55±0.18
Triglycerides (mmol/l)	1.76±0.27	1.75±0.22	1.71±0.25	1.81±0.24
HDL cholesterol (mmol/l)	1.23±0.07	1.23±0.07	1.21±0.06	1.22±0.07
LDL cholesterol (mmol/l)	3.67±0.12	3.82±0.14* ^#^	3.63±0.16	3.52±0.16
Apolipoprotein B (g/l)	1.00±0.04	1.02±0.04^¶^	0.99±0.05	0.97±0.04

* p< 0.01 compared to baseline.

^¶^ p <0.05 and

^#^ p< 0.01 compared to treatment with placebo

**Table 3:** The absolute changes in lipid levels during treatment with Danshen, corrected for the changes during treatment with placebo. CROS analysis according to Senn (see statistical section for explanation). A negative number indicates a decrease by Danshen.

	Treatment effect	95% confidence interval	p-value
Total cholesterol (mmol/l)	0.27	-0.05–0.59	0.089
Triglycerides (mmol/l)	-0.13	-0.50–0.25	0.478
HDL-cholesterol (mmol/l)	-0.02	-0.09–0.06	0.692
LDL-cholesterol (mmol/l)	0.29	0.02–0.55	0.037
ApoB lipoprotein (mmol/l)	0.03	-0.02–0.08	0.167

### Blood pressure

24 h ABPM did not change in response to Danshen treatment (Table 4). The total number of measurements during ABPM was 67±9 of which 17±2 (mean±standard deviation (SD)) during the night with Danshen and 63±10 and 17±2 with placebo treatment, respectively.

**Table 4:** Measurement of Systolic/Diastolic blood pressure before and after treatment with Danshen or placebo. Results are provided as mean±SEM. BP: blood pressure. ABPM: 24-h Ambulatory Blood Pressure Monitoring.

ABPM	Danshen	Placebo
total BP (mmHg)	139±4/89±3	136±4/87±2
day BP (mmHg)	142±4/91±3	139±4/90±2
night BP (mmHg)	130±4/82±2	130±4/79±2

Twenty minute automatic blood pressure measurements were performed prior to placebo and Danshen administration (‘baselines’). Again, both systolic and diastolic blood pressures were not different at baseline (systolic blood pressures before Danshen 140±3 mmHg and before placebo 140±3 mmHg. Corresponding values for diastolic blood pressure were 85±2 mmHg and 85±2 mmHg, respectively).

### Forearm vascular responses to acetylcholine

**Fig 3:**
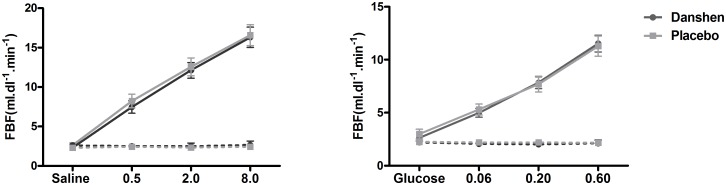
Assessment of endothelial function. Forearm blood flow in response to acetylcholine in the experimental arm (solid lines) and in the non experimental arm (dashed lines) in response to acetylcholine (left panel; dosage 0.5, 2.0, 8.0 μg.dl^-1^.min^-1^) and sodium nitroprusside (right panel; dosage 0.06, 0.20 and 0.60 μg.dl^-1^.min^-1^), during Danshen (dark grey circles) or placebo (light grey squares).

Infusion of acetylcholine induced a dose-dependent increase in FBF in the experimental forearm while FBF did not change in the non-experimental forearm. Danshen treatment did not alter the FBF in response to acetylcholine compared to placebo. With Danshen, FBF increased from 2.3±0.2 ml.dl^-1^.min^-1^ during saline to 7.5±0.8, 12.1±1.0 and 16.3±1.3 ml.dl^-1^.min^-1^ during acetylcholine 0.5, 2.0 and 8.0 μg.dl^-1^.min^-1^ respectively compared to 2.6±0.4 during saline and 8.2±0.9, 12.6± 1.1 and 16.6± 1.3 ml.dl^-1^.min^-1^ during acetylcholine with placebo (Fig 3). Comparable results were obtained when data were expressed as absolute and relative changes in FBF from baseline.

### Forearm vascular responses to sodium nitroprusside

Infusion of sodium nitroprusside induced a dose-dependent increase in FBF in the experimental forearm. FBF did not change in the non-experimental forearm. Treatment with Danshen did not affect vascular responses to sodium nitroprusside. FBF was 2.6±0.3 ml.dl^-1^.min^-1^ during glucose and 5.0± 0.4, 7.8± 0.6 and 11.5± 0.8 ml.dl^-1^.min^-1^ during sodium nitroprusside 0.06, 0.20 and 0.60 μg.dl^-1^.min^-1^, respectively after Danshen treatment, compared to 3.0±0.5 at baseline and 5.3± 0.5, 7.7± 0.7 and 11.3± 1.0 ml.dl^-1^.min^-1^ during nitroprusside after placebo (Fig 3).

### Metabolic and inflammatory parameters

Treatment with Danshen did not affect fasting plasma glucose levels (Danshen 5.7±0.1 mmol/l vs placebo 5.8±0.2 mmol/l, p = 0.48) and HbA1c levels (5.8±0.09% and 5.7±0.08%, respectively, p = 0.45). Also fasting insulin levels, HOMA-IR and HOMA-B did not change during Danshen treatment.

C-reactive protein (CRP) levels were not significantly different after Danshen (2.9±0.7 mg/l) compared to placebo treatment (2.7±0.7 mg/l).

The markers of oxidative stress TBARs and FRAP did not change during Danshen treatment compared to placebo (TBARs 0.09±0.01 vs 0.09±0.01 μmol/mg protein and FRAP 268±11 vs 267±11 μM/mg protein). Furthermore, there were no differences in oxidized LDL, Intercellular Adhesion Molecule (ICAM), Vascular Cell Adhesion Molecule (VCAM) and E-selectin between Danshen and placebo treatment (data not shown). Danshen did not change body weight (data not shown).

### Hemostatic and rheological parameters

Danshen treatment did not change von Willebrand factor antigen levels compared to placebo (112±9% vs 113±9%). The PFA with collagen and epinephrine was 132±7 s when treated with Danshen and 148±7 s when treated with placebo, both within the normal range (p = 0.048 for comparison between Danshen and placebo). Furthermore, the PFA with collagen and ADP was not different between the two treatment periods (97±4 s and 100±4 s, respectively). Taking both PFA tests together, we interpret these data as that Danshen does not relevantly affect platelet function.

**Fig 4:**
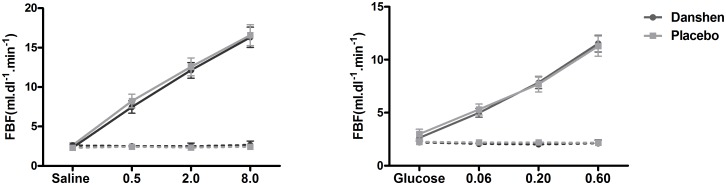
Results of the Nijmegen Hemostasis Assay. A. Thrombin generation. B. Plasmin generation measured by fibrinolysis time.

The coagulation parameters measured by thrombin generation as well as fibrinolysis parameters measured by plasmin generation were not altered by Danshen treatment (Fig 4).

Blood viscosity at a shear rate of 3.16 sˉ¹ was not different after treatment with Danshen (14.4±0.9 mPas*s) compared to placebo (14.2±0.8 mPas*s). However, hematocrit levels were significantly higher after treatment with Danshen (44 l/l vs 43 l/l, p = 0.048). When expressing blood viscosity per l/l of hematocrit we did not find an effect of Danshen on viscosity either.

### TCM-qualification ‘Blood Stasis’

Only two out of the twenty participants (10%) were qualified as having ‘blood stasis’ at inclusion. In both subjects blood stasis was not observed in the following visits. The kappa values, as a measure of between-observer agreement for the diagnosis of blood stasis were 0.19 for TCM visit at baseline, 0.67 for TCM visit after the first treatment period, and -0.08 for TCM visit after the second treatment period. In addition a polychoric correlation was calculated which was 0.43, 0.99 and 0.12 for the first, second and third visit, respectively.

### Side effects and safety

Most reported side effects during Danshen treatment were headache (n = 5), dizziness (n = 3), change in stool frequency (n = 3) and flatulence (n = 2). One serious adverse event occurred during Danshen treatment: a peripheral facial nerve paralysis.

## Discussion

This study did not find beneficial effects of four weeks of treatment with Danshen on cardiovascular risk factors. This is based on the observation that Danshen does not reduce LDL and triglyceride levels and does not decrease blood pressure. Furthermore, Danshen had no effect on endothelial function, markers of inflammation, oxidative stress and glucose metabolism nor on hemostatic and rheological markers.

These results are in contrast with previous studies. A number of explanations may underlie the lack of reduction of cardiovascular risk markers in this study. First, we only used Danshen and not a combination of Chinese herbs.

Tan et al. have shown that a combination of 3 Chinese herbs, including Danshen, decreased fasting triglycerides, cholesterol and non-esterified fatty acids in rats with metabolic syndrome [14]. The Fufang Danshen dripping pill, which is a composite of *Salvia miltiorrhiza*, *Panax notogingseng* and *Cinnamomum camphora*, reduced triglycerides, total cholesterol and LDL cholesterol levels [9].

Second, there are over 80 components of Danshen of which 3 lipophilic and 3 hydrophilic components seem to be the major active components [9, 31]. Our Danshen product was produced via water-extraction method. Possibly, a low concentration in the water-extract of pharmacologically active lipophilic compounds, including tanshinones, led to a lack of efficacy of Danshen. Unfortunately, an alcohol-extract of Danshen is not available in the Netherlands for human use.

Third, the selection of participants was based on evidence-based criteria of increased cardiovascular risk, i.e. increased LDL cholesterol or triglyceride levels and hypertension. The TCM qualification “blood stasis” was only made in 2 out of the 20 participants. Since according to the TCM theory Danshen is used to treat “blood stasis”, advocators of this theory could argue that the selected population might not be suitable to detect any cardiovascular benefit. It is not known what diagnosis according to evidence-based medicine corresponds with this TCM-qualification. Therefore, we cannot exclude the possibility that Danshen is effective in reducing plasma LDL-cholesterol in a subgroup of patients who have “blood stasis” as opposed to the observed increase in plasma LDL-cholesterol in those who are at increased cardiovascular risk according to evidence-based criteria. However, our observed low reproducibility of the observation ‘blood stasis’ and overpresentation of intermediate test results support our decision not to include this TCM-qualification in the inclusion criteria of this trial.

In our study Danshen (water extract) actually increased LDL and total cholesterol levels compared to placebo. This increment in cholesterol levels has also been observed for another food product, namely coffee. It has been shown that boiled, but not filtered, coffee increases total cholesterol and LDL cholesterol [32]. The hypercholesterolemic factor in boiled coffee is retained by the filter paper and eventually this factor was identified as cafestol [33, 34]. Possibly, the method of preparation of Danshen produced or preserved a “hypercholesterolemic factor” that was not produced by methods used by others.

Danshen did not reduce endothelium-dependent vasodilation which suggests that the observed increase in LDL cholesterol of approximately 0.3 mmol/l is too small to have a significant impact on cardiovascular risk. Indeed, endothelium-mediated vasodilation predicts cardiovascular disease and is responsive to changes in plasma cholesterol [35–38]. The Cholesterol Treatment Trialists’ (CTT) Collaboration has shown that each 1 mmol/l reduction in LDL cholesterol reduces the absolute risk of major cardiovascular events with 20% [39]. If we intrapolate these epidemiological observations and assume generalizability of our findings, our observed increase of 0.3 mmol/l in LDL cholesterol would translate into a small increment of 7% in absolute risk of cardiovascular disease. Based on the demographic data of our study population and current cardiovascular risk tables available for this Dutch population, absolute 10-year cardiovascular risk of our subjects to develop a cardiovascular event is 19% for male non-smokers and 35% for male smokers. The water extract of Danshen could theoretically increase these risks to 20.3% and 37.5% for male non-smokers and smokers respectively. However, this projected increase in cardiovascular risk may overestimate the real impact of Danshen on cardiovascular risk, since the increase in LDL-cholesterol was not accompanied by a detectable increase in oxidized LDL cholesterol. Therefore, the impact of Danshen on lipid profile was probably too small to be reflected by a detectable change in acetylcholine-induced forearm vasodilation.

The strength of our study is the randomized, double-blind, placebo controlled design with objective and reproducible endpoints. The cross-over design is potentially limited by carry-over effects. However, we did not detect an order-effect and baseline measurements in blood pressure and plasma lipids did not differ prior to the two treatment periods.

### Conclusion

Four weeks of treatment with the herbal product Danshen (water extract) slightly increased LDL-cholesterol without affecting a wide variety of other risk markers or endothelium-dependent vasodilation, an early marker of vascular injury. These results do not support the use of Danshen water-extract as a single agent to treat risk factors of atherosclerosis.

## Supporting Information

S1 Text The protocol (with final amendements marked as tracked changes) as approved by our ethics committee. (DOCX)

S2 Text Funding, role of funders and conflict of interests. (DOCX)

S3 Text CONSORT Checklist of information to include when reporting a randomised trial. (DOCX)

S4 Text Methods to detect Blood Stasis. (DOCX)

S1 Table Excell file containing the full database for lipids (primary outcome) and blood pressure (secondary outcome). (XLSX)
