# Supplementary material for: Salvia Miltiorrhiza Root Water-Extract (Danshen) Has No Beneficial Effect on Cardiovascular Risk Factors. A Randomized Double-Blind Cross-Over Trial
Source: PLoS One. 2015 Jul 20;10(7):e0128695. doi: 10.1371/journal.pone.0128695 (PMC4508048; doi:10.1371/journal.pone.0128695)
Supplement: S1 Text — (DOCX) [file pone.0128695.s001.docx]

**RESEARCH PROTOCOL**

A double blind, randomized placebo-controlled cross-over study on the cardiovascular effects of *Salvia miltiorrhiza* extract (Danshen)

in patients with hypertension and hyperlipidemia.

Title: A double blind, randomized placebo-controlled cross-over study on the cardiovascular effects of *Salvia miltiorrhiza* extract (Danshen) in patients with hypertension and hyperlipidemia.

| **Protocol ID** | Danshen |
| --- | --- |
| **Short title** | Cardiovascular effects of *Salvia miltiorrhiza* extract (Danshen) |
| **Version** | 6 |
| **Date** | May15^th^ , 2012 |
| **Sponsor** | Prof. F. Russel, PharmD, PhD, pharmacologist  149 Pharmacology - Toxicology  UMC St Radboud  P.O. Box 9101, 6500 HB Nijmegen |
| **Principal Investigator** | Prof. G.A. Rongen, MD, PhD, Internist, Clinical Pharmacologist  149 Pharmacology - Toxicology  UMC St Radboud  P.O. Box 9101, 6500 HB Nijmegen |
| **Associate Investigator**  **(Medical responsibility)** | Prof. C. Tack, MD, PhD, internist  463 Internal Medicine  UMC St Radboud  P.O. Box 9101, 6500 HB Nijmegen |
| **Associate internal investigator** | P.C.M. van Poppel, M.Sc.  463 Internal Medicine  UMC St Radboud  P.O. Box 9101, 6500 HB Nijmegen |
| **Associate external investigators** | K.A. Kruithof, MD, TCM Practitioner  Springweg 7  3511 VH Utrecht  H.H. Tan, MD, TCM Practitioner  Chinees Gezondheidscentrum “Zuidwal”  Wagenstraat 130A  2512 BA Den Haag |
| **Associate investigators**  **(subsidising parties)** | Cinmar Pharma  Contact person(s): J. van der Hoeven, MSc/ K. Groen, PhD |
| **Independent physician(s)** | A.J.M. Rennings, MD  463 Internal Medicine  UMC St Radboud  P.O. Box 9101, 6500 HB Nijmegen |
| **Laboratory sites** | 441 Clinical Chemistry  UMC St Radboud  P.O. Box 9101, 6500 HB Nijmegen |
| **Manufacturing**  **(capsules)** | Basic Pharma Manufacturing BV  Burg. Lemmensstraat 352  6163 JT Geleen |

**PROTOCOL SIGNATURE SHEET**

| **Name** | **Signature** | **Date** |
| --- | --- | --- |
| **Sponsor/Head of Department:**  Prof. Frans Russel, PharmD, PhD  Pharmacologist |  |  |
| **Principal Investigator**  Prof. Gerard Rongen, MD, PhD,  Internist, Clinical Pharmacologist |  |  |
| **Associate Investigator**  **(Medical responsibility)**  Prof. Cees Tack, MD, PhD, internist |  |  |

**TABLE OF CONTENTS**

1. INTRODUCTION AND RATIONALE 10

2. OBJECTIVES 19

3. STUDY DESIGN 20

4. STUDY POPULATION 21

4.1 Population (base) 21

4.2 Inclusion criteria 21

4.3 Exclusion criteria 21

4.4 Diagnosis according to TCM theory 22

4.5 Sample size calculation 22

5. TREATMENT OF SUBJECTS 23

5.1 Investigational product/treatment 23

5.2 Use of co-intervention 24

6. METHODS 25

6.1 Study parameters/endpoints 25

6.1.1 Main study parameters/endpoints 25

6.1.2 Secondary study parameters/endpoints 25

6.1.3 Other study parameters 25

7. SAFETY REPORTING 29

7.1 Section 10 WMO event 29

7.2 Adverse and serious adverse events 29

7.2.1 Suspected unexpected serious adverse reactions (SUSAR) 29

7.2.2 Annual safety report 30

7.3 Follow-up of adverse events 30

7.4 Data Safety Monitoring Board (DSMB) 30

8. STATISTICAL ANALYSIS 31

8.1 Primary analysis 31

8.2 Secondary analysis 31

8.3 Presentation of the results 31

9. ETHICAL CONSIDERATIONS 32

9.1 Regulation statement 32

9.2 Recruitment and consent 32

9.3 Objection by minors or incapacitated subjects (if applicable) 32

9.4 Benefits and risks assessment, group relatedness 32

9.5 Compensation for injury 32

9.6 Incentives (if applicable) 33

10. ADMINISTRATIVE ASPECTS AND PUBLICATION 34

10.1 Handling and storage of data and documents 34

10.2 Amendments 34

10.3 Annual progress report 34

10.4 End of study report 35

10.5 Public disclosure and publication policy 35

11. REFERENCES 36

**LIST OF ABBREVIATIONS AND RELEVANT DEFINITIONS**

**REGULATORY**

| **ABR** | **ABR form (General Assessment and Registration form) is the application form that is required for submission to the accredited Ethics Committee (ABR = Algemene Beoordeling en Registratie)** |
| --- | --- |
| **AE** | **Adverse Event** |
| **AR** | **Adverse Reaction** |
| **CA** | **Competent Authority** |
| **CCMO** | **Central Committee on Research Involving Human Subjects** |
| **CV** | **Curriculum Vitae** |
| **DSMB**  **EFSA** | **Data Safety Monitoring Board**  **European Food Safety Authority** |
| **EU** | **European Union** |
| **EudraCT** | **European drug regulatory affairs Clinical Trials GCP Good Clinical Practice** |
| **IB** | **Investigator’s Brochure** |
| **IC** | **Informed Consent** |
| **IMP** | **Investigational Medicinal Product** |
| **IMPD** | **Investigational Medicinal Product Dossier** |
| **METC** | **Medical research ethics committee (MREC); in Dutch: medisch ethische toetsing commissie (METC)** |
| **(S)AE** | **Serious Adverse Event** |
| **SPC** | **Summary of Product Characteristics (in Dutch: officiële productinfomatie IB1-tekst)** |
| **Sponsor** | **The sponsor is the party that commissions the organisation or performance of the research, for example a pharmaceutical company, academic hospital, scientific organisation or investigator. A party that provides funding for a study but does not commission it is not regarded as the sponsor, but referred to as a subsidising party.** |
| **SUSAR**  **VWA** | **Suspected Unexpected Serious Adverse Reaction**  **Voedsel en Waren Authoriteit** |
| **Wbp** | **Personal Data Protection Act (in Dutch: Wet Bescherming Persoonsgevens)** |
| **WMO**  **SCIENTIFIC**  **CVD**  **eNOS**  **FMD**  **HDL(-C)**  **hsCRP**  **IPC**  **IR**  **LC-MS**  **LDL(-C)**  **NO**  **ROS**  **SNP**  **TBARS**  **TCM**  **TG**  **QUICKI** | **Medical Research Involving Human Subjects Act (Wet Medisch-wetenschappelijk Onderzoek met Mensen**  **CardioVascular Disease**  **endothelial Nitric Oxide Synthase**  **Flow Mediated Dilatation**  **High Density Lipoprotein Cholesterol**  **C-reactive protein, measured by use of a high-sensitivity assay**  **Ischemic PreConditioning**  **Ischemic Reperfusion Injury**  **Liquid Chromatography-Mass Spectometry**  **Low Density Lipoprotein Cholesterol**  **Nitric Oxide**  **Reactive Oxygen Species**  **Sodium nitroprusside**  **ThioBarbituric Acid Reactive Substances**  **Traditional Chinese Medicine**  **triglycerides**  **Quantitative insulin-sensitivity check index** |

**SUMMARY**

**Rationale:** Extracts of the plant *Salvia miltiorrhiza* (Chinese name “Danshen”) have been used as traditional Chinese medicine in the treatment of cardiovascular diseases, such as angina pectoris and myocardial infarction. In China, it has been extensively studied in clinical trials over the last decades. However, the methodological quality of these studies was poor, and reliable conclusions from these studies could not be drawn. Several preclinical studies point towards promising effects of Danshen on risk factors of atherosclerotic cardiovascular diseases, such as hyperlipidemia and hypertension. Danshen has potent antioxidant properties and is shown to have promising effects on endothelial dysfunction and ischemia-reperfusion injury. Therefore, we want to investigate the cardiovascular effects of *Salvia miltiorrhiza* extract (Danshen) in a well-controlled clinical study.

**Objective:** Our primary objective is to determine the effect of Salvia miltiorrhiza extract (Danshen) on hyperlipidemia. Secondary objective is to investigate the effect of Danshen on hypertension. Further objectives are to determine its effect on endothelial dysfunction, and to explore its effect on markers for oxidative stress and on inflammatory activation of the vascular system and adipose tissue , and on hemorheological parameters and hemostasis, and on insulin sensitivity. These objectives will primarily be evaluated for all patients in the study. In a secondary analysis, the effects will be evaluated in a subgroup of patients that have the diagnosis “blood stasis” according to Traditional Chinese Medicine theory.

**Study design:** Single-centre, placebo-controlled, randomized, double-blind, cross-over study.

**Study population:** subjects with hypertension and hyperlipidemia, aged 40 – 70 years.

**Intervention:** The study will consist of two treatment periods of four weeks with a washout period of four weeks. Total duration 12 weeks. In one treatment period, Danshen (oral capsules, administered twice daily) will be given, in the other treatment period, a similar dosing schedule with placebo capsules will be used.

**Main study parameters/endpoints:** Outcome parameters will be assessed in the 4^th^ week of each treatment period. To study the effect of Danshen on hyperlipidemia, in particular LDL-cholesterol, but also HDL-cholesterol, total cholesterol, total triglycerides, apolipoprotein B plasma levels and LDL oxidation will be measured. Blood pressure will be quantified by 24-h Ambulatory Blood Pressure Monitoring and office blood pressure monitoring (during 20 min. period). Endothelial function will be assessed by both the forearm vasodilator response to infusion of acetylcholine and nitroprusside into the brachial artery, quantified by venous occlusion strain gauge plethysmography. Furthermore, plasma biomarkers of oxidative stress (e.g. TBARS) and vascular inflammation (e.g. hsCRP) will be sampled. Inflammatory activation of adipose tissue will be assessed by morphology (e.g. fat cell size, influx of macrophages), histochemistry, Q-PCR and protein analysis (e.g. IL-1β, IL-6, adiponectin).

In addition, markers of hemostasis (e.g. von Willebrand factor), and hemorheological parameters, such as blood viscosity, will be determined. Insulin sensitivity will be assessed by use of the QUICKI index.

**Nature and extent of the burden and risks associated with participation, benefit and group relatedness:** *Salvia miltiorrhiza* extract (Danshen) has been used for hundreds of years by large populations. No serious side effects have been described. The cannulation of the brachial artery (under local anaesthesia), and the venous blood samples will give some local discomfort. 24-h Ambulatory Blood Pressure Monitoring will give some discomfort during sleep. Plethysmography will cause temporary and completely reversible numbness and discomfort in both hands due to inflation of the wrist-cuffs. The adipose tissue biopsy is preceded by infiltration with local anaesthetics, but may induce a hematoma. The subjects may benefit from participating in this study when Danshen appears to improve vascular function and risk factors. A subject fee is to be provided.

# INTRODUCTION AND RATIONALE

Cardiovascular Disease (CVD) is the leading cause of death worldwide. Mortality due to CVD has declined as a result of both primary (general population without a history of CVD) and secondary prevention (patients with a history of CVD) initiatives. The best approach to the problem is the treatment and prevention of risk factors for CVD. Hypertension and hyperlipidemia belong to the main risk factors of the development of atherosclerotic cardiovascular disease (1-6).

Danshen is the dried root extract of the annual sage plant *Salvia miltiorrhiza*. In China, Danshen is an extremely popular traditional medicinal herb for the prevention and treatment of (atherosclerotic) cardiovascular diseases, such as angina pectoris and myocardial infarction (7;8). It has a market that exceeded US $ 120 million in 2002 (9). According to Traditional Chinese Medicine (TCM) theory, Danshen is used in patients to treat “blood stasis” as it “moves blood” and promotes the flow of “qi” (i.e. the energy in the meridians). As medicinal herb, Danshen is used orally or even intravenously either alone or (mostly) in combination with other herbs.

Up to now, more than 70 compounds have been isolated and structurally identified from Danshen. The major components are hydrophilic salvianolic acids and lipophilic diterpenoids, known as tanshinones (10). The effects of Danshen (compounds) on cardiovascular disease have been investigated extensively in both animal and human studies. Preclinical *in vitro* and *in vivo* experiments suggest positive effects of Danshen on hyperlipidemia. For example, Sieveking et al. showed that when acetylated LDL-loaded human monocyte derived macrophages (HMDMs) were exposed to Danshen and Gegen, the accumulation of free cholesterol and total cholesteryl esters in these HMDMs significantly decreased (11). In another study, SK0506, a Danshen containing preparation, significantly reduced fasting plasma triacylglycerol and non-esterified fatty acids and cholesterol in high-fat diet-fed rats (12). Ji et al. showed that Danshen treatments significantly prevented the detrimental effects of oxidized LDL on endothelial progenitor cell functions and decreased the lipid peroxidation end product malondialdehyde (13). Furthermore, the Danshen compounds Salvianolic acid A and B, tanshinone IIA and magnesium tanshinoate B were shown to inhibit LDL oxidation (13-17).

Of interest, other studies showed positive effects of Danshen compounds on hypertension(18;19). For example, oral tanshinone significantly reduced mean arterial pressure from 161.2 ± 6.9 to 130.0 ± 7.8 mmHg in hypertensive hamsters (18). Furthermore, other preclinical studies also showed promising effects of Danshen compounds on endothelial function (by stimulation of endothelial nitric oxide synthase and subsequent increase of nitric oxide concentration) (18;20), ischemia and ischemia-reperfusion injury (via several actions, such as by antioxidant activity and anti-inflammatory effects, for example by inhibition of the expression of adhesion molecules and by decreasing blood C-reactive protein) (21-25), and on hemorheological parameters, such as blood viscosity (26). Also insulin-sensitizing activities of tanshinones have been described (27).

Many randomized controlled trials were conducted to evaluate the efficacy of Danshen on ischemic vascular diseases. These clinical cardiovascular studies with Danshen are also promising, but unfortunately, the majority of these studies are not well-controlled (9;28;29). The conclusion of the most recent meta-analyses (including Cochrane analysis) on the use of Danshen preparations for acute myocardial infarction, for acute ischemic stroke, and for angina pectoris is that the effect of Danshen products for the treatment of these cardiovascular diseases is promising but not proven, and that more studies are needed to proof its efficacy (28) (30-32). In particular, the authors suggested that future trials should assure adequate concealment of allocation, blinding of outcome assessors, and use of functional outcome as the primary outcome measured at long-term follow up. Therefore, we aim to start a well-controlled trial to investigate the effects of Danshen in atherosclerotic cardiovascular diseases. Actually, we will address the following hypothesis:

**The use of Danshen will be an effective approach in the primary prevention of atherosclerotic cardiovascular diseases by reducing one or more risk factors, i.e. by significantly lowering plasma lipids and/or blood pressure in patients with hyperlipidemia and hypertension.**

*Endothelial dysfunction*

Vascular endothelium plays a pivotal role in modulating vascular tone, vessel diameter, and blood flow in response to humoral, neural and mechanical stimuli by synthesizing and releasing vasoactive substances. Endothelial dysfunction, broadly defined, occurs when the endothelium fails to serve its normal physiologic and protective mechanisms. The presence of intact endothelium is essential for acetylcholine (ACh) to induce dilatation of isolated arteries. In contrast, when the endothelium is removed, the arteries constrict in response to ACh. ACh stimulates the release of a potent vasodilating substance from the endothelium, identified as nitric oxide (NO), originally identified as endothelium-derived relaxing factor (EDRF). Nitric oxide is the major endothelium-derived vasodilating substance. Other endothelium-derived vasodilators include prostacyclin and bradykinin. Prostacyclin acts synergistically with NO to inhibit platelet aggregation. Bradykinin stimulates release of NO, prostacyclin, endothelium-derived hyperpolarizing factor, another vasodilator, and tissue plasminogen activator (t-PA), thereby most likely playing a role in fibrinolysis. The endothelium also produces vasoconstrictor substances, such as endothelin and angiotensin II (33;34).

Damage to the endothelium upsets the balance between vasodilation and vasoconstriction and initiates a number of events/processes that promote or exacerbate atherosclerosis. Decreased production or activity of NO, manifested as impaired vasodilation, may be one of the earliest signs of atherosclerosis. When NO is lost, the normal vasodilator response to ACh is replaced by paradoxical constriction resulting from the direct effect of ACh on vascular smooth muscle. NO los can occur after mechanical denudation of the endothelium or due to pathological disease states affecting the endothelium. It might also occur as a result of metabolic toxins, such as free fatty acids (FFAs) and inflammatory cytokines, including IL-6 and TNF-α. Metabolic abnormalities might alter endothelial intracellular signaling pathways, such as the PI3K–Akt pathway. Finally, reactive oxygen species (ROS), particularly superoxide, might scavenge vascular NO and prevent it from inducing vascular smooth muscle relaxation (33;35).

NO is generated by conversion of the amino acid L-arginine to NO and L-citrulline by the enzyme nitric oxide synthase (NOS). There are three major isoforms of NOS, encoded by separate genes on separate chromosomes: neuronal NOS (nNOS), or type 1 NOS; inducible NOS (iNOS), or type 2 NOS; and eNOS, or type 3 NOS. The isoform eNOS is constitutively expressed by the endothelium. NO exerts its relaxing effect on vascular smooth muscle by activation of guanylate cyclase leading to increased production of cyclic guanosine monophosphate (cGMP) and a reduction in intracellular calcium (33;34).

*Endothelial dysfunction in relation to hyperlipidemia*

Disorders in lipoprotein metabolism (dyslipidemia or hyperlipidemia) can result in premature atherosclerosis. Dyslipidemias can be classified as hypercholesterolemia (i.e. elevated levels of total cholesterol and low density lipoprotein cholesterol), hypertriglyceridemia, combined hyperlipidemia, and low levels of high density lipoprotein cholesterol (HDL-C). Both elevated levels of low density lipoprotein cholesterol (LDL-C) and decreased levels of HDL-C predispose to premature atherosclerosis (36;37). A recent meta-analysis found that 1 mmol/L lower total cholesterol was associated with about a half, a third and a sixth lower ischemic heart disease mortality in both men and women at ages 40-49, 50-69 and 70-89 years, respectively (5). In addition, hypertriglyceridemia has been associated with cardiovascular disease (CVD) as well (38). The first meta-analysis, published in 1996, clearly showed a significant, independent, but modest effect in overall CVD risk per 1 mmol/L increase of triglycerides (TGs) (39). Patients with combined hyperlipidemia are at increased risk of developing atherosclerosis and coronary heart disease (40).

The pathophysiology of combined hyperlipidemia may include hepatic overproduction of apolipoprotein (apo) B-100 or TGs, resulting in the increased secretion of very-low-density lipoprotein (VLDL). Alternatively, it may involve impaired catabolism of lipoproteins including TG-rich lipoproteins or cholesterol-rich LDL (41).

Enhanced LDL cholesterol and particularly oxidized LDL play an important role in endothelial dysfunction (42-44). Direct effects on the NO signaling pathway involving protein kinase C and G proteins may contribute to the detrimental effects of oxidized LDL on endothelial function. Lysophosphatidylcholine, a specific component of oxidized LDL, mimics the effects of oxidized LDL on endothelial function and may be the responsible mediator. Hypercholesterolemia also promotes generation of superoxide within the vessel wall, in part by enhancing the activity of NAD(P)H and xanthine oxidases, and can thereby indirectly impair NO bioavailability (33) (and references herein).

Unlike other lipoproteins which are atherogenic, the HDLs are unique in that they appear to be vasculoprotective and antiatherogenic. HDL is believed to play a key role in the process of reverse cholesterol transport, in which it promotes the efflux of excess cholesterol from peripheral tissues and returns it to the liver for biliary excretion (45). HDL particles appear to oppose atherogenesis by inhibiting endothelial cell adhesion molecule and selectin expression, stimulating endothelial nitric oxide and prostacyclin production, inhibiting endothelial cell apoptosis, decreasing platelet aggregability and inhibiting LDL oxidation (37).

*Endothelial dysfunction in relation to hypertension*

The vascular regulatory site for the control of blood pressure by adjustment of total peripheral resistance resides mainly in arterioles. The pathogenesis of hypertension has been associated with the function of endothelial nitric oxide (NO) synthase (eNOS), the enzyme that plays a crucial role in the regulation of vessel diameter. eNOS catalyzes the production of NO, which in turn mediates arteriolar vasodilatation, reduces peripheral resistance, and lowers the blood pressure. Thus alterations in the function of this enzyme can lead to elevation of blood pressure. In fact, experimental studies showed that animals lacking this enzyme demonstrate hypertension (46;47).

*Oxidative stress and endothelial dysfunction*

Both excess generation of reactive oxygen species (ROS) including superoxide anion and oxidized LDL cholesterol (oxLDL) and decreased antioxidant defense mechanisms contribute to enhanced degradation of NO (48;49). Superoxide radical (O_2_^-^) accumulates as a result of excess mitochondrial lipid oxidation. NO is the kinetically preferred scavenger for O_2_^-^ because scavenging by NO occurs at an extremely rapid rate; three times faster than the interaction with other antioxidants (i.e. superoxide dismutase). The reaction between O_2_^-^ and NO not only contributes to loss of NO bioavailability for endothelial function, but it also results in formation of peroxynitrite (ONOO^-^), itself a potent oxidant. Furthermore, O_2_^-^ and ONOO^-^ can oxidize tetrahydrobiopterin (BH_4_), a cofactor necessary for normal production of NO by eNOS, to the BH_3_ radical, which leads to eNOS uncoupling. Uncoupled eNOS produces O_2_^-^ instead of NO, thus resulting in a vicious cycle. Taken together, oxidative stress results in reduced NO bioavailability via a combination of direct elimination and decreased production of NO. The reduced bioavailability of NO in turn compromises all the anti-atherogenic functions of the endothelium (48;50).

*Inflammation of the vascular system and endothelial dysfunction*

Inflammation plays an important pathogenic role in atherosclerosis. By promoting inflammation within the vessel wall, dysfunctional endothelium sets the stage for both initiation and progression of atherosclerotic lesions. The production of cytokines, such as TNF-α and IL-6, and expression of cellular adhesion molecules, such as VCAM-1, ICAM-1 and E-selectin, will be enhanced, when the endothelium becomes dysfunctional. Adhesion molecules play a crucial role in the interaction of the endothelial surface with circulating leukocytes and mediate the recruitment of leukocytes and their accumulation in the intima of the vessel wall (51;52). *In vitro*, expression of cellular adhesion molecules was induced by C-reactive protein (CRP), a component of the acute phase response (53). Elevated serum levels of CRP have been widely considered to be nonspecific but sensitive as a marker of acute inflammatory response promoting atherosclerosis (53).

*Inflammatory activation of adipose tissue and* *endothelial dysfunction*

Adipose tissue is an important secretory organ that produces various bioactive substances known as adipocytokines, including leptin, TNF- α, and adiponectin, which contribute to obesity-linked metabolic and vascular diseases. In tissue cultures, adiponectin attenuated monocyte attachment to endothelial cells by reducing the expression of adhesion molecules on endothelial cells. Adiponectin also suppressed lipid accumulation in monocyte-derived macrophages through the suppression of macrophage scavenger receptor expression. These *in vitro* data suggested the anti-atherogenic properties of adiponectin. In mice, adiponectin was shown to modulate proinflammatory reactions in the vascular wall. Clinically, hypoadiponectinemia has been identified in patients with obesity, type 2 diabets, and coronary artery disease (54;55).

*Haemostasis and endothelial dysfunction*

Under physiological conditions, the vascular endothelium produces many substances that contribute importantly to haemostasis, fibrinolysis, and regulation of vessel tone and permeability. One such substance is the multimeric glycoprotein von Willebrand factor (vWF), which is produced almost exclusively by endothelial cells by a variety of stimuli, such as hypoxia, inflammatory cytokines, thrombin, leukocyte elastase, histamine, endotoxin, adrenaline and vasopressin. Plasma levels of vWF are raised in different states of endothelial damage and have therefore been proposed as useful markers of endothelial dysfunction. vWF plays a crucial role in platelet adhesion and aggregation under high shear conditions and also functions as a stabilizing carrier protein of coagulation factor VIII (56;57).

The vulnerable plaque typically contains a prominent accumulation of inflammatory cells including macrophages and T lymphocytes. Macrophages can weaken the connective tissue framework of the fibrous cap of atheromatous plaques by secreting extracellular matrix degrading enzymes and thereby facilitate plaque rupture. Proinflammatory actions of dysfunctional endothelium potentially play an important role in converting stable atheromatous plaques in unstable plaques prone to rupture. The functional state of the endothelium may also affect the consequences of plaque rupture. If plaque rupture occurs, prevention of superimposed thrombus formation, a critical step underlying acute coronary syndromes, depends on successful inhibition of platelet aggregation and coagulation and on activation of fibrinolysis. NO is an important mediator in these defense mechanisms. Reduced NO bioavailability due to endothelial dysfunction may therefore facilitate thrombus formation. Dysfunctional endothelium also leads to increased production of plasminogen activator inhibitor–1, an inhibitor of fibrinolysis, and reduced production of the fibrinolytic component tissue plasminogen activator contributing to a thrombogenic state. By losing its protective properties and allowing the unopposed action of atherogenic factors on the vessel wall, dysfunctional endothelium is a major promoter of both atherogenesis and thrombosis and, consequently, cardiovascular events (33).

*Toxicology and safety data of Salvia miltiorrhiza extract (Danshen)*

Traditionally, Danshen has been considered to be relatively non-toxic, which was supported by preclinical toxicological studies. LD_50_ of Danshen Glucose Injection (DGI) was 26.9 g/kg for iv and 36.1 g/kg for intraperitoneal (ip) administration, respectively, in mice, and 27.0 g/kg for iv administration in SD rats (58). The maximum tolerated dosage of supercritical CO_2_ extract of Fufang Danshen (Danshen, *Panax* *notoginseng* and Borneol) was 10 g/kg with ig administration in mice. The result was 970 times above the clinical dosage (59). An experiment on chronic toxicity of DGI revealed that there were no pathological changes to be observed at a dose of 0.8 and 2.0 g/kg, although aspartate aminotransferase (AST) was increased, and plasma albumin was significantly reduced at a dose of 2.0 g/kg, which was 2.5 times clinical dosage, after iv administration in Beagle dogs for 180 days. At a dose of 5.0 g/kg, blood coagulation time was prolonged, AST was increased, albumin was reduced significantly, and some pathological changes were observed in liver, such as hepatic steatosis and edema, after 180 days iv administration. All these changes returned to normal after 21 days of drug withdrawal (60). Chronic toxicity of supercritical CO_2_ extract of Compound Danshen was reported in SD rats after ig administration at a dosage of 0.195, 0.391 and 0.782 g/kg, respectively, which were equivalent to 20.2, 40.3 and 80.6 times, respectively, of the clinical dosage. In the 0.195 g/kg dose group, no toxicity was observed. In the two higher dose groups, some microscopic pathological changes were detected in the liver, and blood alanine aminotransferase and alkaline phosphatase were increased [31]. More studies on mice bone marrow cell micronucleus test, the mice sperm abnormality test, Ames test, and teratogenicity tests of Danshen were carried out. None of mutagenicity, maternity toxicity, embryo toxicity or teratogenicity was found (61).

In general, the incidence of adverse drug reactions of *Salvia miltiorrhiza* is quite low. However, it should be mentioned that it is not common use (in China) to systematically report adverse events of herbal products. Most patients do not readily disclose their use of herbs to their health care providers, and physicians may not routinely ask about such use (62). Despite underreporting, many clinical studies with Danshen containing products, that reported adverse events, were performed and so far, no serious adverse events related to *Salvia miltiorrhiza* have been reported (9;30-32). The most common adverse drug reactions reported in clinical studies of oral *Salvia miltiorrhiza* doses were mild gastrointestinal reaction, upper abdominal discomfort, thirsty feeling, headache, dizziness, bad appetite, skin rash and itching (9).

Although serious adverse events of Danshen are not known, usage of Danshen can lead to interactions with other drugs. *In vitro* studies in human liver microsomes and *in vivo* studies in rodents and in humans have shown that Danshen or its components may have an effect on the activity of cytochrome P450 enzymes (CYP), as was shown for CYP3A4, CYP1A2, CYP2D6, CYP2C9, and CYP2C11 (63-66). Thus far, interactions between oral Danshen and CYP3A4 substrates seem to be of clinical relevance (65). In addition, *in vitro* studies in MDCKII-MDR1 monolayers showed that cryptotanshinone and tanshinone IIA and IIB are a substrate of P-glycoprotein (MDR1; P-gp) (67-69). Also *in vivo* in *Mdr1a* knockout mice it was shown that tanshinone IIB is a substrate of P-gp (69). However, the clinical relevance of interactions between Danshen and other P-gp substrates has not been studied yet.

*Registration and marketing of Salvia miltiorrhiza extract (Danshen)*

In China, a number of pharmaceutical dosage forms of *Salvia miltiorrhiza,* that have been registered at the Chinese State of Food and Drug Administration (SFDA), are commercially available. For example, Danshen Tablet produced by SINE Laboratories (registration number Z20055235 and Danshen Tablet produced by Furen Medicine Group (registration number Z20073092).There is a variety of formulations of *Salvia miltiorrhiza*, including tablets, capsules, dripping pills, oral liquids, sprays and injectables. The Fufang Danshen Tablet and Fufang Danshen Dripping Pill (the Chinese word “fufang” means a combination preparation) are the two most frequently used products in China and have been officially listed in the Chinese pharmacopoeia. The Fufang Danshen Dripping Pill, which is a composite of *Salvia miltiorrhiza*, *Panax notoginseng*, and either *borneol* or *Cinnamomum camphora*, has also been registered as a prescription drug in several countries, including Vietnam, South Korea, Pakistan, India and the United Arab Emirates. It was the first TCM product approved for phase II clinical trials by the Food and Drug Administration (FDA) in the United States in 1997 (IND No. 56956). Recently, it has successfully completed Phase II clinical trials in the US and will soon begin Phase III investigations (9).

In the European Union (EU) marketing of herbal products has been regulated under national legislation for years, leading to differences in legal status of these herbal products. In one member state, a product may be regulated as a food supplement, while in the other member state the same product is considered a medicinal product. New legislation for traditional herbal medicinal products (EC/2004/24) has been set in the EU to improve harmonization providing free movement of these products in the inner market. This new legislation came into force March 2004. A transition period of 7 years for already marketed products was foreseen. This transition period ended on April 1^st^ , 2011 (70). As a consequence, herbal products can only be marketed in Europe as a herbal medicinal product (claiming a specific indication) by following a marketing authorization procedure. This requires a full dossier with preclinical and clinical data to prove safety and efficacy (70).

Like most herbal products, Danshen is marketed in the Netherlands as a food supplement, which is under the control of VWA (Voedsel en Warenauthoriteit) and EFSA (European Food Safety Authority) (European Regulation (EC) No 1924/2006). Thereby, Danshen is available for patients on prescription by a certified acupuncturist/herbalist, who can order it at a wholesaler or specialized pharmacy (e.g. NatuurApotheek).

As documented safety/toxicology data of Danshen are available, we expect that no additional preclinical safety testing will be required for marketing of Danshen in Europe as a herbal medicinal product (70). However, first we will perform a proof-of-principle study, in which we want to explore whether oral treatment with Danshen as a single herb is an effective strategy in influencing favorable risk factors for CVD, i.e. in the primary prevention of CVD. Therefore, we will make use of *Salvia miltiorrhiza* extract (Danshen), which is produced by Kaiser Pharmaceuticals Co. (KPC) in Taiwan, and which is commercially available in the Netherlands via its European distributor, Sinecura NV and subsequently via a specialized pharmacy, such as NatuurApotheek (Pijnacker). Using this extract, Basic Pharma Manufacturing BV, a contract manufacturing company, will produce Danshen capsules and placebo capsules. When results of this clinical pilot study are positive, i.e. when Danshen treatment significantly lowers plasma lipids and/or blood pressure in patients with hyperlipidemia or hypertension, Cinmar Pharma is going to develop a Danshen tablet, consisting of *Salvia miltiorrhiza* extract which is cultured and produced in the Netherlands under Good Agricultural Practice conditions. In the latter case, a marketing authorization procedure shall be followed, and clinical (dose-finding) studies with the product of Cinmar will be performed.

# OBJECTIVES

**Primary Objective:**

- To determine the effect of *Salvia miltiorrhiza* extract (Danshen) on LDL-cholesterol in patients with hypertension and hyperlipidemia. In addition, we want to determine the effect of *Salvia miltiorrhiza* extract (Danshen) on other lipids, in particular total cholesterol, total triglycerides, HDL-cholesterol, and apolipoprotein B, in patients with hypertension and hyperlipidemia.

**Secondary Objective:**

- To determine the effect of *Salvia miltiorrhiza* extract (Danshen) on blood pressure in patients with hypertension and hyperlipidemia.

**Other Objectives:**

- To investigate the effect of *Salvia miltiorrhiza* extract (Danshen) on endothelial function in patients with hypertension and hyperlipidemia.
- To determine the effect of *Salvia miltiorrhiza* extract (Danshen) on plasma markers of oxidative stress, in particular TBARS, antibodies against oxidized LDL, and ferric-tripyridyltriazine complex, in patients with hypertension and hyperlipidemia.
- To determine the effect of *Salvia miltiorrhiza* extract (Danshen) on vascular inflammation and inflammatory activation of adipose tissue, in particular inflammatory cytokines IL-6, IL-8, TNF-α, hsCRP, adiponectin and the expression of vascular adhesion molecules VCAM-1, ICAM-1, and E-selectin, in patients with hypertension and hyperlipidemia.
- To determine the effect of *Salvia miltiorrhiza* extract (Danshen) on hemostasis, in particular coagulation and fibrinolysis, platelet aggregation, von Willebrand factor, and haemorheological parameters, in particular blood viscosity, in patients with hypertension and hyperlipidemia.
- To determine the effect of *Salvia miltiorrhiza* extract (Danshen) on insulin sensitivity in patients with hypertension and hyperlipidemia.

These objectives will primarily be evaluated in the total study population. In a secondary analysis, the primary and secondary objectives will be evaluated in the subgroup of patients that have the diagnosis “blood stasis” according to Traditional Chinese Medicine theory.

# STUDY DESIGN

Twenty subjects suffering from hypertension and hyperlipidemia without cardiovascular disease and without anticoagulant and/or lipid-lowering medication will be selected by advertisements. Study enrollment comprises inclusion by a Western physician. After inclusion, two Traditional Chinese Medicine practitioners will in addition also diagnose the patients according to Traditional Chinese Medicine theory before the start of the study (i.e. before randomization) and at the end of both treatment periods (as described in Chapter 4: Study population). The study population will be stratified by age and by the subtype “blood stasis”.

After signing of the informed consent and study enrollment/randomization, the subjects will receive a four week treatment with Danshen capsules (4 capsules of 500 mg granulate, three times daily, 1 hour after breakfast and dinner) and a four week treatment with placebo in a randomized order and with a wash out period of four weeks in between. Treatment period 1 thus continues from days 1 to 28 (week 1 to 4), treatment period two from days 57 to 84 (week 9 to 12). A compliance check will be performed by telephone call 2 weeks after the start of each treatment period, i.e. on day 14 and day 70. The measurements (as described in Chapter 7: Methods) will be performed before each treatment period, at the end of the four-week treatment in week 4 after an overnight fasting and 24 hour caffeine abstinence. In week 12 all measurements of week 4 are repeated (See Figure 1 for a schematic overview).


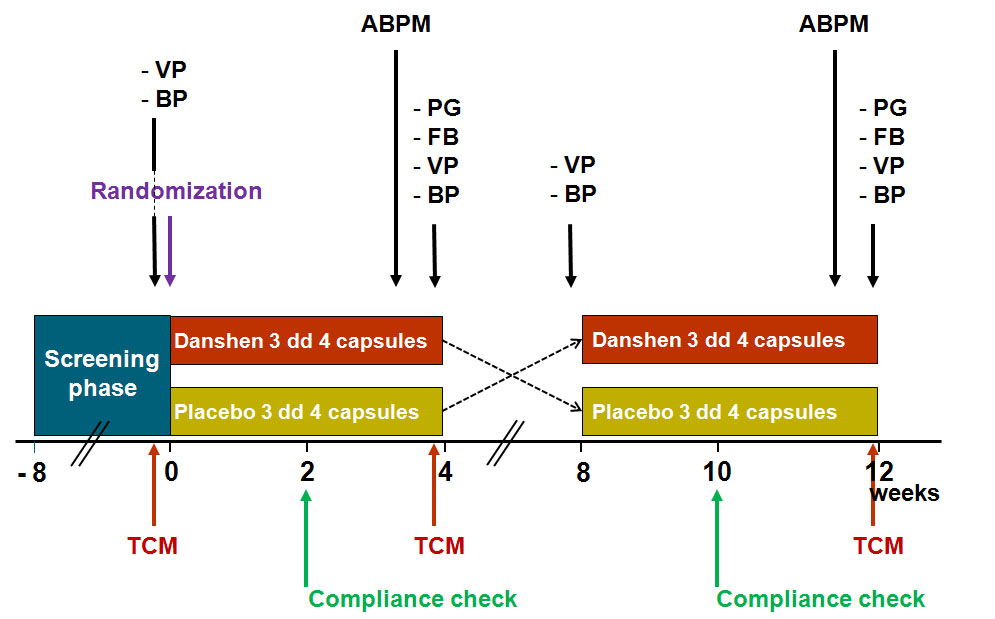


*Figure 1. Schematic design of the double-blinded, randomized, placebo-controlled cross-over study.*

*VP: venapuncture; BP: blood pressure; ABPM: 24-h Ambulatory Blood Pressure Monitoring; PG: Phlethysmography; FB: fat biopsy; TCM: TCM diagnosis.*

# STUDY POPULATION

## Population (base)

Men and women (50%/50%) aged between 40 and 70 suffering from hyperlipidemia and hypertension. The study population will be stratified by age and by the subtype “blood stasis”.

## Inclusion criteria

- Age: 40-70
- Women:
  - postmenopausal, or
  - use of contraceptive pill (start of Danshen/placebo treatment shall be concomitant with start of contraceptive treatment)
- Hyperlipidemia:
  - elevated level of triglycerides: > 1.7 mmol/L
  - elevated level of LDL-cholesterol: > 3.5 mmol/L
- Hypertension:
  - systolic pressure > 140 mm Hg
  - diastolic pressure > 90 mm Hg
- Signed informed consent

## Exclusion criteria

- Alcohol or drug abuse
- History of cardiovascular disease (myocard infarct, angina pectoris, CVA)
- Diabetes mellitus, when treated with insulin
- Pregnancy
- Concomitant (chronic) use of:

*Medicinal products:*

- - ACE-inhibitors, including a.o. captopril, enalapril, ramipril
  - AT_1_-antagonists, including a.o. losartan, valsartan, irbesartan
  - Statins, including a.o. simvastatin, rosuvastatin
  - Anticoagulant drugs, including a.o. aspirin
  - Calciumantagonists (amlodipine, diltiazem, felodipine, lercanidipine, nifedipine, nisoldipine, nitrendipine, verapamil)
  - > 1 antihypertensive drug
  - High-dose antihypertensive medication (above defined daily dose)
  - Drugs which are exclusively metabolised by CYP3A4 (Flockhart DA; P450 drug interaction table), including for example erythromycin, midazolam, cyclosporine, HIV antivirals

*Food products:*

- - (Antioxidant) vitamin supplements
  - Other herbs, including a.o. St Janskruid
  - Grapefruit juice
- Hyperlipidemia which needs conventional treatment
  - elevated level of triglycerides: > 8 mmol/L
  - elevated level of LDL-cholesterol: > 5 mmol/L
- Hypertension which needs conventional treatment:
  - systolic pressure > 180 mm Hg
  - diastolic pressure > 110 mm Hg
- Clinically significant liver disease (3 times the upper normal limit of ALAT,ASAT)
- Clinically significant anemia (male Hb < 6,9 mmol/L, female < 6,25 mmol/L)
- Abnormal creatinine clearance defined as MDRD < 60 ml/min/1.73m^2^
- Participation to any drug-investigation during the previous 90 days
- Use of any herbal product during the previous 30 days

## Diagnosis according to TCM theory

According to TCM theory, Danshen is used to treat “blood stasis”. Two TCM practitioners will independently diagnose the patients for “blood stasis” by pulse and tongue diagnosis and interrogation (see Methods section). A patient will only be classified with the subtype “blood stasis” when this is diagnosed by both TCM practitioners.

## Sample size calculation

*Primary analysis*

Our primary objective of the study is to determine the effect of *Salvia miltiorrhiza* extract (Danshen) on LDL-cholesterol in patients with hypertension and hyperlipidemia. In Azar et al, the effect of ezetimibe/atorvastatin therapy on LDL cholesterol level was a reduction of approximately 0.3 mmol/L compared with placebo/atorvastatin therapy, with a standard deviation of approximately 0.45. The correlation between the measurements of the two periods is unknown, but when it is 0.7, 20 subjects are sufficient to detect a slightly smaller difference of 0.225 mmol/L with 80% power. When the correlation is 0.5, the detectable difference is approx. 0.3 mmol/L. Although no information about the correlation is available, a study with 20 evaluable patients seems adequate to detect relevant differences of 0.225 to 0.3 mmol/L (71).

*Secondary analysis*

The number of patients that have blood stasis according to TCM criteria may be less than 20. However, the analysis on this subgroup of patients is only secondary and exploratory.

# TREATMENT OF SUBJECTS

## Investigational product/treatment

20 subjects will use Danshen capsules (4 capsules of 500 mg granulate, three times daily) for 4 weeks

20 subjects will use placebo capsules (4capsules, three daily) for 4 weeks

*Salvia miltiorrhiza* extract (Danshen) is produced by Kaiser Pharmaceuticals Co. (KPC) in Taiwan, and is commercially available in the Netherlands via its European distributor, Sinecura NV, and subsequently via a specialized pharmacy such as NatuurApotheek (Pijnacker) (as a herbal product, which is under the control of VWA and EFSA). Using this extract, Basic Pharma Manufacturing BV, a contract manufacturing company, will produce Danshen capsules and placebo capsules. The capsules will be stored at the Clinical Research Centre Nijmegen (CRCN) in a closed, temperature controlled room in containers until usage.

Briefly, the production process of Danshen extract is as follows: first, a water decoction of Salvia root is made. Subsequently, the water extract is 5 times concentrated via standard distillation procedure. After the concentration procedure, a drying and granulation procedure is followed using a fluid bed spray dryer. For granulation starch is used as carrier (50%). Then, the granules are filtered, which results in a final bulk product consisting of granules of a specified size. Finally, the bulk product is filled into containers (final product). The granulation process contributes to the homogeneity of the Danshen powder/extract.

According to the German Pharmacopeia (Arzneibuch der Chinesischen Medizin, monografien des Arzneibuches der Volksrepublik China 2000 und 2005), the recommended dosage of Danshen root is 9 -15 g. Each capsule contains 500 mg granulate consisting of both 50% (water)extract of Danshen and 50% starch. The root – extract ratio is 1:5, thus, a dosage of 3 dd 4 capsules with 500 mg granulate is equal to 3 dd 4 capsules with 250 mg extract (3 dd 1 g extract), which is equal to 3 dd 5 g root (thus 15 g root daily).

Both in the Chinese and German Pharmacopoeia an assay is described for the determination of the content of tanshinone IIA and salvianolic acid B in Radix et Rhizoma Salvia Miltiorrhizae (Danshen root). Danshen root (not extract!) should contain not less than 0.2% of tanshinone IIA and not less than 3.0% of salvianolic acid B. There are no specifications available for the content of these or other compounds in Danshen extract. Therefore, we will determine the content of tanshininone IIA and salvianolic acid B in the Danshen capsules before administration to the patient.

The 20 subjects will receive a four week treatment with Danshen capsules and a four week treatment with placebo and with a wash out period of four weeks in between. The subjects will be randomized in a double-blind way, in such a way that 10 subjects will receive Danshen capsules in treatment period 1 and placebo in treatment period 2 and 10 subjects will receive placebo in treatment period 1 and Danshen capsules in treatment period 2 (See Figure 1). On site, only the clinical trial manager or delegate will have access to the randomization list and will provide the investigational product for each subject according to the predefined randomization list. The investigational product will be provided in containers. To check for compliance the patients will be asked to maintain a notebook.

The treatment code in the randomization list must not be broken except in medical emergencies when the appropriate management of the patient necessitates knowledge of the treatment randomization. The investigator(s) must document date, time and reason for unblinding and report to the Drug Safety Officer any breaking of the treatment code as soon as possible. UMC St Radboud reserves the right to break the code for any SAE that is unexpected and is suspected to be causally related to the investigational product. Treatment codes will not be broken for the planned analyses until all decisions on the evaluability of data from each individual patient have been made and documented (i.e. after database lock).

## Use of co-intervention

Acetylcholine

Sodium nitroprusside

# METHODS

## Study parameters/endpoints^[[1]](#footnote-1)^

### Main study parameters/endpoints

1. *Hyperlipidemia*

Blood will be drawn in fasting state by venapuncture to measure the primary outcome parameter LDL-cholesterol. The additional parameters, total cholesterol, total triglycerides, HDL-cholesterol, and apolipoprotein B will be measured in a similar way.

### Secondary study parameters/endpoints

1. *Hypertension*

Blood pressure (BP) will be quantified by 24-h Ambulatory Blood Pressure Monitoring (ABPM) by using a fully automated device (SpaceLabs Medical Inc., Redmond USA) in week 4 (at home). Using this device BP will be measured every 15 minutes between 0700 and 2300 h and every 30 min during night-time (72). Furthermore, office blood pressure will be monitored (during 20 minutes one measurement every two minutes, resulting in n=10 per day) in week 4 at the day of measurements (in clinic) (see below). This scheme will be repeated in the second treatment period.

### Other study parameters

1. *Endothelial dysfunction*

Endothelial dysfunction is a common denominator for a variety of changes the endothelium undergoes during atherogenesis. In recent years a number of tests has been developed in order to quantify endothelial function. The use of these tests is widespread, since coronary endothelial dysfunction is a useful marker for predicting future cardiovascular events. Endothelial function in these tests is assessed by demonstrating (lack of) nitric oxide mediated vasodilatation in an artery. Due to the invasive nature of coronary endothelial function testing, attention has shifted to forearm vasoreactivity, which is less invasive.

At present, the most feasible and robust technique for testing endothelial function in peripheral circulation is:

Measurement of the forearm blood flow (FBF; ml/100ml forearm volume/min.) by **strain-gauge plethysmography**, after local intra-brachial infusion of NO-agonists, such as acetylcholine (ACh), a stimulator of endogenous NO production, and sodium nitroprusside, an exogenous NO-donor.

Using venous strain gauge plethysmography, we will determine the forearm blood flow (FBF) response to increasing concentrations of acetylcholine and sodium nitroprusside. Subjects will be asked to present fasting and to abstain from caffeine containing food and beverages during the preceding 24 h. Patients should lie down during the whole experiment.

A wrist cuff, an upperarm cuff and a mercury-filled silastic strain gauge will be applied to both arms. A 27 G needle will be inserted in the brachial artery of the non-dominant arm to administer the vasodilating agents during plethysmography. A flowchart of the procedure is shown in Figure 2. The vasodilatory response to acetylcholine (ACh) and sodium nitroprusside (SNP) will be assessed successively. During preparation, the volume of the non-dominant arm will be measured by water displacement to adjust the concentration of the vasodilating agent to forearm volume.

Vasodilating agents will be administered in three increasing doses during 5 minutes a dose. The agents are considered to have reached a stable concentration within 3 minutes. Forearm blood flow (FBF) will therefore be assessed during the last 2 minutes of each dose. A 30 minute pause is made prior to each agent, to ensure proper baseline measurements. Blood flow to the hands will be occluded by inflation of the wrist cuffs to 300 mm Hg and restored during NaCl infusion. The upper arm cuffs are repeatedly inflated by a Hokanson rapid cuff inflator (Hokanson, Belllevue, US) to a pressure of 40 mm Hg to occlude venous return for 10 seconds during FBF registration. One cycle of FBF measurements will consist of approximately 8 measurements a dose. The total procedure will take approximately three hours.


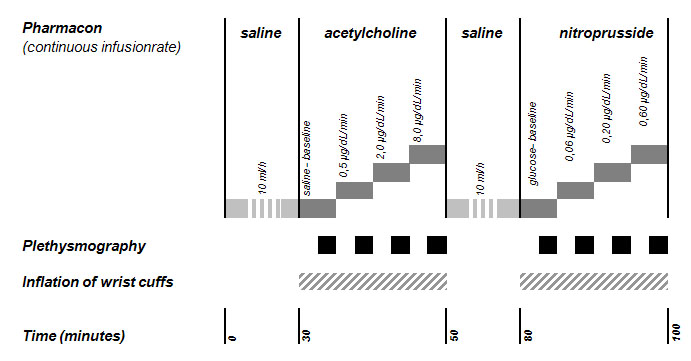


*Figure 2. Schematic overview of venous strain gauge plethysmography procedure*

1. *Markers of oxidative stress*

Blood will be drawn by venapuncture at the end of week 4 and week 12 to measure IgG and IgM antibodies against oxidized LDL by enzyme-linked immunosorbent assay (ELISA) as described by van Tits et al. (73). In addition, lipid peroxidation will be determined by measuring thiobarbituric acid reactive substances (TBARS) in plasma using a fluorimetric assay (73). Furthermore, antioxidant capacity will be determined by means of ferric reducing ability of plasma assay (FRAP) (74;75).

1. *Vascular inflammation*

Indicators of vascular inflammation (soluble vascular cell adhesion molecule-1 (sVCAM-1), soluble intercellular adhesion molecule-1 (sICAM-1), E-selectin, hsCRP, IL-6, IL-8 and TNF-α will be determined at the end of week 4 and week 12 as well, again material is collected by venous blood sampling. The concentrations of sVCAM-1 and sICAM-1 will be determined by a multiplex cytokine assay.

1. *Inflammatory activation of adipose tissue*

A subcutaneous fat biopsy, a so called mini liposuction, will be taken after an overnight fast at the end of week 4 and week 12. After application of local anaesthesia adipose tissue will be aspirated with a 14 gauge stainless steel needle from the lateral aspect of the hypogastrium, inferior to the umbilicus.

Inflammatory status of adipose tissue will be assessed by morphology (fat cell size, influx of macrophages, determination of crown-like structures), histochemistry (macrophage and CD68 staining), Q-PCR (expression of relevant target genes, including IL-1beta, IL-6, caspase-1, PPARy, adiponectin) and protein analysis (including IL-1beta, IL-6, IL-8, adiponectin, TNFalpha).

1. *Markers of haemostasis and hemorheological parameters*

Blood will be drawn by venapuncture at the end of week 4 and week 12. Anticoagulant activity and fibrinolytic activity will be determined by use of the validated Nijmegen Haemostasis Assay, which measures both thrombin generation and plasmin generation simultaneously by fluorimetric detection (76). The effect on platelet aggregation will be determined by a standardized platelet function analysis based on shear stress (PFA-100) (77). The concentration of von Willebrand factor (vWF) antigen will be determined by PFA-100 and by standard ELISA (78).

Blood viscosity will be determined with a golden standard technique, i.e. a Contraves LS 30 Couette viscometer (79).

1. *Insulin sensitivity*

A fasting blood sample will be drawn by venapuncture at the end of week 4 and week 12 to measure blood glucose and insulin levels. Subsequently, the quantitative insulin sensitivity check index (QUICKI = 1/[log(I_0_) + log(G_0_)]) will be determined (80;81).

1. *Safety parameters*

Considering safety, all subjects will be screened after inclusion by means of a complete medical history, physical examination and laboratory evaluation. For the latter blood will be drawn by venapuncture. An EKG will be produced to search for and exclude subjects with specific conduction abnormalities. Biochemistry and hematology will be (partly) repeated during both treatment periods.

Laboratory evaluation for safety parameters comprises: Hb, Ht, thrombocytes, leucocytes, creatinine, potassium, ASAT, ALAT, bilirubin (total, direct), albumin

1. *Serum concentration of Danshen compounds*

Blood will be drawn by venapuncture at the end of week 4 and week 12. Serum levels of cryptotanshinone, danshensu, rosmarinic acid, salvianolic acid B, tanshinone I, tanshinone IIA, and dihydrotanshinone will be determined by liquid chromatographic-mass spectrometry (LC-MS) (10;82-84).

1. *Subtyping according to Traditional Chinese Medicine theory*

According to TCM theory, Danshen is used to treat “blood stasis”. Two TCM practitioners will independently diagnose the patients for “blood stasis” by pulse and tongue diagnosis and interrogation. A patient will only be classified with the subtype “blood stasis” when this is diagnosed by both TCM practitioners.

*Pulse diagnosis*

The typical pulse for blood stasis is choppy but powerful, which is caused by the low mobility of blood or by the lack of thin fluid substances within the blood. However, as blood stasis usually occurs in combination with other syndromes, there may be a deep, wiry, tense or slow pulse, depending on the concomitant syndrome.

*Tongue diagnosis*

Dark blue or purple dots on the tongue with a purple shading of the tongue body generally indicates blood stasis. The location of the blood stasis in the body can be determined from the region of the tongue affected. E.g. when the tongue tip has a purple-bluish color or is dark, this indicates heart blood stasis. Furthermore, when the sublingual veins are dark blue or deep purple, or crooked, then in most cases blood stasis is present.

# SAFETY REPORTING

Although safety reporting is related to investigational medicinal products, and Salvia miltiorrhiza extract (Danshen) is a food product, we will follow the procedures for safety reporting of IMPs (with the exception of EudraCT procedure and informing Medicines Evaluation Board (MEB); in the latter case we will inform Voedsel en WarenAutoriteit (VWA), if applicable)

## Section 10 WMO event

In accordance to section 10, subsection 1, of the WMO, the investigator will inform the subjects and the reviewing accredited METC if anything occurs, on the basis of which it appears that the disadvantages of participation may be significantly greater than was foreseen in the research proposal. The study will be suspended pending further review by the accredited METC, except insofar as suspension would jeopardise the subjects’ health. The investigator will take care that all subjects are kept informed.

## Adverse and serious adverse events

Adverse events are defined as any undesirable experience occurring to a subject during a clinical trial, whether or not considered related to the investigational food product. All adverse events reported spontaneously by the subject or observed by the investigator or his staff will be recorded and concomitant medication will be checked.

A serious adverse event is any untoward medical occurrence or effect that at any dose results in death;

- is life threatening (at the time of the event);
- requires hospitalisation or prolongation of existing inpatients’ hospitalisation;
- results in persistent or significant disability or incapacity;
- is a congenital anomaly or birth defect;
- is a new event of the trial likely to affect the safety of the subjects, such as an unexpected outcome of an adverse reaction, lack of efficacy of an IMP used for the treatment of a life threatening disease, major safety finding from a newly completed animal study, etc.

All SAEs will be reported to the accredited METC that approved the protocol, according to the requirements of that METC, and to VWA (Voedsel en WarenAutoriteit)

### Suspected unexpected serious adverse reactions (SUSAR)

Adverse reactions are all untoward and unintended responses to an investigational product related to any dose administered.

Unexpected adverse reactions are adverse reactions, of which the nature, or severity, is not consistent with the applicable investigational food product information as known from literature and described in this protocol (Introduction, section “Toxicology and safety data of *Salvia miltiorrhiza* extract (Danshen)” .

The sponsor will report expedited the following SUSARs to the METC, CCMO and to VWA (Voedsel en WarenAutoriteit):

- SUSARs that have arisen in the clinical trial that was assessed by the METC;
- SUSARs that have arisen in other clinical trial of the same sponsor and with the same investigational product, and that could have consequences for the safety of the subjects involved in the clinical trial that was assessed by the METC.

The remaining SUSARs are recorded in an overview list (line-listing) that will be submitted once every half year to the METC. This line-listing provides an overview of all SUSARs from the study medicine, accompanied by a brief report highlighting the main points of concern.

The expedited reporting will occur not later than 15 days after the sponsor has first knowledge of the adverse reactions. For fatal or life threatening cases the term will be maximal 7 days for a preliminary report with another 8 days for completion of the report.

### Annual safety report

In addition to the expedited reporting of SUSARs, the sponsor will submit, once a year throughout the clinical trial, a safety report to the accredited METC, and to VWA (Voedsel en WarenAutoriteit)

This safety report consists of:

- a list of all suspected (unexpected or expected) serious adverse reactions, along with an aggregated summary table of all reported serious adverse reactions, ordered by organ system, per study;
- a report concerning the safety of the subjects, consisting of a complete safety analysis and an evaluation of the balance between the efficacy and the harmfulness of the medicine under investigation.

## Follow-up of adverse events

All adverse events will be followed until they have abated, or until a stable situation has been reached. Depending on the event, follow up may require additional tests or medical procedures as indicated, and/or referral to the general physician or a medical specialist.

## Data Safety Monitoring Board (DSMB)

Not applicable.

# STATISTICAL ANALYSIS

## Primary analysis

All analyses will be according to the per-protocol principle. Only patients who sufficiently complied with the trial’s protocol (i.e. after inclusion > 90% of the prescribed dosages (Danshen capsules and placebo capsules) is taken) will be considered in the analysis.

*Primary outcome*

LDL-cholesterol will be analyzed in a linear model with the sequence of the treatments as covariate.

The additional parameters, total cholesterol, total triglycerides, HDL-cholesterol, and apolipoprotein B, will be analyzed in a similar way.

*Secondary outcome*

The mean of the blood pressure measurements in week 4 will be evaluated in a linear model with the sequence of the treatments as covariate.

*Other outcomes*

Other variables will also be evaluated in a linear model with the sequence of the treatments as covariate. When variables are skewed, a logarithmic transformation will be used. Whenever appropriate, a random effects model will be used to account for repeated measurements.

## Secondary analysis

The analysis of the primary and secondary parameters will be repeated in the TCM “blood stasis” subgroup, i.e. the group of all patients that have “blood stasis” according to both TCM experts. Also this analysis will be according to the per-protocol principle. The outcome parameters are determined in a similar way as described above.

## Presentation of the results

Ninety five percent confidence intervals will be presented.

# ETHICAL CONSIDERATIONS

## Regulation statement

This study will be conducted according to the principles of the Declaration of Helsinki (2004) and in accordance with the Medical Research Involving Human Subjects Act (WMO) and international Good Clinical Practice guidelines.

## Recruitment and consent

Participants will be recruited from the general population by means of advertisements in local or regional newspapers. The advertisement text has been added to this application.

Informed consent will be signed before screening. Prior to this, extensive information will be given by means of the patient information brochure. An oral summary of the most important topics (e.g. the risks, the lack of benefit with regard to personal health and the right to withdraw without consequences) will be given before screening, including time for additional questions. If necessary, additional time to (re)consider participation will be provided.

## Objection by minors or incapacitated subjects (if applicable)

Not applicable.

## Benefits and risks assessment, group relatedness

Side effects of Salvia miltiorrhiza extract tend to be very mild. Plethysmography will cause numbness and discomfort in both hands due to inflation of the wrist-cuffs. This is temporarily and completely reversible. Finally, bruising may occur after venapuncture or removal of the intra-arterial 27 G cannula. Measures like pressure bandage will be taken to minimise the risk. 24-h Ambulatory Blood Pressure Monitoring will give some discomfort during sleep. The adipose tissue biopsy is preceded by infiltration with local anesthetics, but may induce a hematoma. Subjects will not benefit much from participating in this study. An advantage may be that the subject is exposed to a putative beneficial treatment. If this treatment appears to be effective, the participant can continue this treatment because the Salvia miltiorrhiza-extract is freely available in the market. A subject fee is to be provided.

## Compensation for injury

A liability insurance is effected with Market Forum in London. In case of damage, participants can apply to Akkermans van Elten Assurantiën BV, postbus 181, 6660 AD Elst. This insurance is in accordance with the legal requirements in the Netherlands (Article 7 WMO and the Measure regarding Compulsory Insurance for Clinical Research in Humans of 23th June 2003) and provides cover for damage to research subjects through injury or death caused by the study.

1. € 450.000,-- (i.e. four hundred and fifty thousand Euro) for death or injury for each subject who participates in the Research;
2. € 3.500.000,-- (i.e. three million five hundred thousand Euro) for death or injury for all subjects who participate in the Research;
3. € 5.000.000,-- (i.e. five million Euro) for the total damage incurred by the organisation for all damage disclosed by scientific research for the Sponsor as ‘verrichter’ in the meaning of said Act in each year of insurance coverage.

The insurance applies to the damage that becomes apparent during the study or within 4 years after the end of the study.

## Incentives (if applicable)

A subject fee of € 400,- is to be provided. This fee will be paid fully when the participant completed the study or has to be withdrawn prematurely because of side effects. When withdrawn for other reasons, subjects will receive a fee proportional to the time of participation.

# ADMINISTRATIVE ASPECTS AND PUBLICATION

## Handling and storage of data and documents

For all participating subjects, a cumulative medical research file will be archived at the Clinical Research Centre Nijmegen (CRCN). This file will never leave the Radboud University Nijmegen Medical Centre. This source document can only be viewed by trial monitors and investigators involved in trials for which the subject has signed an informed consent. If for some unexpected reason, the volunteer needs medical attention, physicians who are not involved in this research will only be allowed to have insight in the medical research file after additional written informed consent by the volunteer or his representative.

The following data will be archived in the source document: copy of the signed informed consent form, study code, medical history, physical examination, EKG at screening, registration of visits (including tablet count and possible adverse events) and printed laboratory results.

In addition to the source document, a case report form (CRF) will be completed. The CRF is anonymised and may leave the hospital for data management or monitoring purposes. A copy of the CRF remains at CRCN for 15 years.

The following data will be archived in the CRF: subject code, screening data, flow charts of all visits (including the procedure and data of forearm blood flow measurements), tablet counts, adverse events (including a summary) and results of lab tests. The subject code log will be archived in a locked site at CRCN.

## Amendments

A ‘substantial amendment’ is defined as an amendment to the terms of the METC application, or to the protocol or any other supporting documentation, that is likely to affect to a significant degree:

- the safety or physical or mental integrity of the subjects of the trial;
- the scientific value of the trial;
- the conduct or management of the trial; or
- the quality or safety of any intervention used in the trial.

All substantial amendments will be notified to the METC and to the competent authority.

Non-substantial amendments will not be notified to the accredited METC and the competent authority, but will be recorded and filed by the sponsor.

## Annual progress report

The sponsor/investigator will submit a summary of the progress of the trial to the accredited METC once a year. Information will be provided on the date of inclusion of the first subject, numbers of subjects included and numbers of subjects that have completed the trial, serious adverse events/ serious adverse reactions, other problems, and amendments.

## End of study report

The sponsor will notify the accredited METC and the competent authority of the end of the study within a period of 90 days. The end of the study is defined as the last patient’s last visit.

In case the study is ended prematurely, the sponsor will notify the accredited METC and the competent authority within 15 days, including the reasons for the premature termination.

Within one year after the end of the study, the investigator/sponsor will submit a final study report with the results of the study, including any publications/abstracts of the study, to the accredited METC and the Competent Authority.

## Public disclosure and publication policy

Results will be submitted for publication to a peer reviewed medical journal as soon as possible. The protocol summary will be made public on [www.clinicaltrial.gov](http://www.clinicaltrial.gov). CINMAR Pharma shall have the right to review each publication and presentation (including, but not limited to, full papers, abstracts, poster presentations and oral presentations) of results of the Study prior to its submission to anyone not affiliated with CINMAR Pharma or the Sponsor. A copy of each proposed publication and presentation shall be submitted to CINMAR Pharma for review at least fifteen (30) days (or ten (15) days in the case of abstracts and full papers, posters presentations and oral presentations not exceeding two (2) double spaced pages in length) prior to such submission. The Sponsor and the Principal Investigator acknowledge that such right is for the purpose of enabling CINMAR Pharma to provide peer input regarding the scientific content and conclusions of such publications and presentations, to provide the Principal Investigator with information which may not have been previously provided or to ensure that none of CINMAR Pharma’s confidential Information will be disclosed and to create the opportunity to timely prepare a patent application.

# REFERENCES

(1) Rosamond W, Flegal K, Friday G, Furie K, Go A, Greenlund K, et al. Heart disease and stroke statistics--2007 update: a report from the American Heart Association Statistics Committee and Stroke Statistics Subcommittee. Circulation 2007 Feb 6;115(5):e69-171.

(2) Gluckman TJ, Baranowski B, Ashen MD, Henrikson CA, McAllister M, Braunstein JB, et al. A practical and evidence-based approach to cardiovascular disease risk reduction. Arch Intern Med 2004 Jul 26;164(14):1490-500.

(3) O'Keefe JH, Carter MD, Lavie CJ. Primary and secondary prevention of cardiovascular diseases: a practical evidence-based approach. Mayo Clin Proc 2009 Aug;84(8):741-57.

(4) Lewington S, Clarke R, Qizilbash N, Peto R, Collins R. Age-specific relevance of usual blood pressure to vascular mortality: a meta-analysis of individual data for one million adults in 61 prospective studies. Lancet 2002 Dec 14;360(9349):1903-13.

(5) Lewington S, Whitlock G, Clarke R, Sherliker P, Emberson J, Halsey J, et al. Blood cholesterol and vascular mortality by age, sex, and blood pressure: a meta-analysis of individual data from 61 prospective studies with 55,000 vascular deaths. Lancet 2007 Dec 1;370(9602):1829-39.

(6) Ezzati M, Lopez AD, Rodgers A, Vander HS, Murray CJ. Selected major risk factors and global and regional burden of disease. Lancet 2002 Nov 2;360(9343):1347-60.

(7) Cheng TO. Cardiovascular effects of Danshen. Int J Cardiol 2007 Sep 14;121(1):9-22.

(8) Lin TH, Hsieh CL. Pharmacological effects of Salvia miltiorrhiza (Danshen) on cerebral infarction. Chin Med 2010;5:22.

(9) Zhou L, Zuo Z, Chow MS. Danshen: an overview of its chemistry, pharmacology, pharmacokinetics, and clinical use. J Clin Pharmacol 2005 Dec;45(12):1345-59.

(10) Li YG, Song L, Liu M, Hu ZB, Wang ZT. Advancement in analysis of Salviae miltiorrhizae Radix et Rhizoma (Danshen). J Chromatogr A 2009 Mar 13;1216(11):1941-53.

(11) Sieveking DP, Woo KS, Fung KP, Lundman P, Nakhla S, Celermajer DS. Chinese herbs Danshen and Gegen modulate key early atherogenic events in vitro. Int J Cardiol 2005 Oct 20;105(1):40-5.

(12) Tan Y, Kamal MA, Wang ZZ, Xiao W, Seale JP, Qu X. Chinese herbal extracts (SK0506) as a potential candidate for the therapy of the metabolic syndrome. Clin Sci (Lond) 2011 Apr;120(7):297-305.

(13) Ji KT, Chai JD, Xing C, Nan JL, Yang PL, Tang JF. Danshen protects endothelial progenitor cells from oxidized low-density lipoprotein induced impairment. J Zhejiang Univ Sci B 2010 Aug;11(8):618-26.

(14) O K, Lynn EG, Vazhappilly R, Au-Yeung KK, Zhu DY, Siow YL. Magnesium tanshinoate B (MTB) inhibits low density lipoprotein oxidation. Life Sci 2001 Jan 12;68(8):903-12.

(15) Shiao MS, Chiu JJ, Chang BW, Wang J, Jen WP, Wu YJ, et al. In search of antioxidants and anti-atherosclerotic agents from herbal medicines. Biofactors 2008;34(2):147-57.

(16) Niu XL, Ichimori K, Yang X, Hirota Y, Hoshiai K, Li M, et al. Tanshinone II-A inhibits low density lipoprotein oxidation in vitro. Free Radic Res 2000 Sep;33(3):305-12.

(17) Zhao GR, Zhang HM, Ye TX, Xiang ZJ, Yuan YJ, Guo ZX, et al. Characterization of the radical scavenging and antioxidant activities of danshensu and salvianolic acid B. Food Chem Toxicol 2008 Jan;46(1):73-81.

(18) Kim DD, Sanchez FA, Duran RG, Kanetaka T, Duran WN. Endothelial nitric oxide synthase is a molecular vascular target for the Chinese herb Danshen in hypertension. Am J Physiol Heart Circ Physiol 2007 May;292(5):H2131-H2137.

(19) Leung SW, Zhu DY, Man RY. Effects of the aqueous extract of Salvia Miltiorrhiza (danshen) and its magnesium tanshinoate B-enriched form on blood pressure. Phytother Res 2010 May;24(5):769-74.

(20) Tang Y, Garg H, Geng YJ, Bryan NS. Nitric oxide bioactivity of traditional Chinese medicines used for cardiovascular indications. Free Radic Biol Med 2009 Sep 15;47(6):835-40.

(21) Sun J, Huang SH, Tan BK, Whiteman M, Zhu YC, Wu YJ, et al. Effects of purified herbal extract of Salvia miltiorrhiza on ischemic rat myocardium after acute myocardial infarction. Life Sci 2005 Apr 29;76(24):2849-60.

(22) Han JY, Fan JY, Horie Y, Miura S, Cui DH, Ishii H, et al. Ameliorating effects of compounds derived from Salvia miltiorrhiza root extract on microcirculatory disturbance and target organ injury by ischemia and reperfusion. Pharmacol Ther 2008 Feb;117(2):280-95.

(23) Xia WJ, Yang M, Fok TF, Li K, Chan WY, Ng PC, et al. Partial neuroprotective effect of pretreatment with tanshinone IIA on neonatal hypoxia-ischemia brain damage. Pediatr Res 2005 Oct;58(4):784-90.

(24) O'Brien KA, Ling S, Abbas E, Dai A, Zhang J, Wang WC, et al. A Chinese Herbal Preparation Containing Radix Salviae Miltiorrhizae, Radix Notoginseng and Borneolum Syntheticum Reduces Circulating Adhesion Molecules. Evid Based Complement Alternat Med 2008 Sep 27.

(25) Xu G, Zhao W, Zhou Z, Zhang R, Zhu W, Liu X. Danshen extracts decrease blood C reactive protein and prevent ischemic stroke recurrence: a controlled pilot study. Phytother Res 2009 Dec;23(12):1721-5.

(26) Hou WC, Tsay HS, Liang HJ, Lee TY, Wang GJ, Liu DZ. Improving abnormal hemorheological parameters in aging guinea pigs by water-soluble extracts of Salvia miltiorrhiza Bunge. J Ethnopharmacol 2007 May 22;111(3):483-9.

(27) Jung SH, Seol HJ, Jeon SJ, Son KH, Lee JR. Insulin-sensitizing activities of tanshinones, diterpene compounds of the root of Salvia miltiorrhiza Bunge. Phytomedicine 2009 Apr;16(4):327-35.

(28) Yu S, Zhong B, Zheng M, Xiao F, Dong Z, Zhang H. The quality of randomized controlled trials on DanShen in the treatment of ischemic vascular disease. J Altern Complement Med 2009 May;15(5):557-65.

(29) Adams JD, Wang R, Yang J, Lien EJ. Preclinical and clinical examinations of Salvia miltiorrhiza and its tanshinones in ischemic conditions. Chin Med 2006;1:3.

(30) Wu B, Liu M, Zhang S. Dan Shen agents for acute ischaemic stroke. Cochrane Database Syst Rev 2004;(4):CD004295.

(31) Wu T, Ni J, Wu J. Danshen (Chinese medicinal herb) preparations for acute myocardial infarction. Cochrane Database Syst Rev 2008;(2):CD004465.

(32) Jia Y, Huang F, Zhang S, Leung SW. Is danshen (Salvia miltiorrhiza) dripping pill more effective than isosorbide dinitrate in treating angina pectoris? A systematic review of randomized controlled trials. Int J Cardiol 2011 Jan 18.

(33) Behrendt D, Ganz P. Endothelial function. From vascular biology to clinical applications. Am J Cardiol 2002 Nov 21;90(10C):40L-8L.

(34) Huang PL. eNOS, metabolic syndrome and cardiovascular disease. Trends Endocrinol Metab 2009 Aug;20(6):295-302.

(35) Davignon J, Ganz P. Role of endothelial dysfunction in atherosclerosis. Circulation 2004 Jun 15;109(23 Suppl 1):III27-III32.

(36) Rahilly-Tierney CR, Lawler EV, Scranton RE, Gaziano JM. Cardiovascular benefit of magnitude of low-density lipoprotein cholesterol reduction: a comparison of subgroups by age. Circulation 2009 Oct 13;120(15):1491-7.

(37) Toth PP. Drug treatment of hyperlipidaemia: a guide to the rational use of lipid-lowering drugs. Drugs 2010 Jul 30;70(11):1363-79.

(38) Stalenhoef AF, de GJ. Association of fasting and nonfasting serum triglycerides with cardiovascular disease and the role of remnant-like lipoproteins and small dense LDL. Curr Opin Lipidol 2008 Aug;19(4):355-61.

(39) Hokanson JE, Austin MA. Plasma triglyceride level is a risk factor for cardiovascular disease independent of high-density lipoprotein cholesterol level: a meta-analysis of population-based prospective studies. J Cardiovasc Risk 1996 Apr;3(2):213-9.

(40) Goldstein JL, Schrott HG, Hazzard WR, Bierman EL, Motulsky AG. Hyperlipidemia in coronary heart disease. II. Genetic analysis of lipid levels in 176 families and delineation of a new inherited disorder, combined hyperlipidemia. J Clin Invest 1973 Jul;52(7):1544-68.

(41) Contacos C, Barter PJ, Sullivan DR. Effect of pravastatin and omega-3 fatty acids on plasma lipids and lipoproteins in patients with combined hyperlipidemia. Arterioscler Thromb 1993 Dec;13(12):1755-62.

(42) Mertens A, Holvoet P. Oxidized LDL and HDL: antagonists in atherothrombosis. FASEB J 2001 Oct;15(12):2073-84.

(43) Galle J, Hansen-Hagge T, Wanner C, Seibold S. Impact of oxidized low density lipoprotein on vascular cells. Atherosclerosis 2006 Apr;185(2):219-26.

(44) Diaz MN, Frei B, Vita JA, Keaney JF, Jr. Antioxidants and atherosclerotic heart disease. N Engl J Med 1997 Aug 7;337(6):408-16.

(45) Lewis GF, Rader DJ. New insights into the regulation of HDL metabolism and reverse cholesterol transport. Circ Res 2005 Jun 24;96(12):1221-32.

(46) Huang PL, Huang Z, Mashimo H, Bloch KD, Moskowitz MA, Bevan JA, et al. Hypertension in mice lacking the gene for endothelial nitric oxide synthase. Nature 1995 Sep 21;377(6546):239-42.

(47) Shesely EG, Maeda N, Kim HS, Desai KM, Krege JH, Laubach VE, et al. Elevated blood pressures in mice lacking endothelial nitric oxide synthase. Proc Natl Acad Sci U S A 1996 Nov 12;93(23):13176-81.

(48) Munzel T, Gori T, Bruno RM, Taddei S. Is oxidative stress a therapeutic target in cardiovascular disease? Eur Heart J 2010 Nov;31(22):2741-8.

(49) Tsimikas S, Bergmark C, Beyer RW, Patel R, Pattison J, Miller E, et al. Temporal increases in plasma markers of oxidized low-density lipoprotein strongly reflect the presence of acute coronary syndromes. J Am Coll Cardiol 2003 Feb 5;41(3):360-70.

(50) Wallace JP, Johnson B, Padilla J, Mather K. Postprandial lipaemia, oxidative stress and endothelial function: a review. Int J Clin Pract 2010 Feb;64(3):389-403.

(51) Willerson JT, Ridker PM. Inflammation as a cardiovascular risk factor. Circulation 2004 Jun 1;109(21 Suppl 1):II2-10.

(52) Corrado E, Rizzo M, Coppola G, Fattouch K, Novo G, Marturana I, et al. An update on the role of markers of inflammation in atherosclerosis. J Atheroscler Thromb 2010 Feb;17(1):1-11.

(53) Pasceri V, Willerson JT, Yeh ET. Direct proinflammatory effect of C-reactive protein on human endothelial cells. Circulation 2000 Oct 31;102(18):2165-8.

(54) Matsuda M, Shimomura I, Sata M, Arita Y, Nishida M, Maeda N, et al. Role of adiponectin in preventing vascular stenosis. The missing link of adipo-vascular axis. J Biol Chem 2002 Oct 4;277(40):37487-91.

(55) Ouchi N, Ohishi M, Kihara S, Funahashi T, Nakamura T, Nagaretani H, et al. Association of hypoadiponectinemia with impaired vasoreactivity. Hypertension 2003 Sep;42(3):231-4.

(56) Spiel AO, Gilbert JC, Jilma B. von Willebrand factor in cardiovascular disease: focus on acute coronary syndromes. Circulation 2008 Mar 18;117(11):1449-59.

(57) Blann AD. Assessment of endothelial dysfunction: focus on atherothrombotic disease. Pathophysiol Haemost Thromb 2003 Sep 20;33(5-6):256-61.

(58) Yang JR, Gui C, Xiong Y, Chen GX, Ding BP, Song JG. Study on acute and topical toxicity of Danshen Glucose Injection. Acta Academiae Medicinae Wannan 2007;26(1):11-4.

(59) Zhang XJ, Wang S, Wang GQ. The studies on toxicology of compound Dan Shen extract liquid. Pharmacology and Clinics of Chinese Materia Medica 2005;21(5):55-7 (in Chinese).

(60) Yang JR, Ding BP, Chen GX, Gui CQ, Fu WS, Xu CY, et al. Study on a long-term toxicity test of Danshen glucose injection in Beagle dog. Acta Academiae Medicinae Wannan 2005;24(1):14-9 (in Chinese).

(61) Jiang ZhR, Jing MW, Ge YJ, Liu KL, Ou ShP, Yu MZh, et al. Mutagenicity and teratogenicity tests of Danshen tea. Teratogenesis and Mutagenesis 2004;2004:232-4 (in Chinese).

(62) Tachjian A, Maria V, Jahangir A. Use of herbal products and potential interactions in patients with cardiovascular diseases. J Am Coll Cardiol 2010 Feb 9;55(6):515-25.

(63) Qiu F, Zhang R, Sun J, Jiye A, Hao H, Peng Y, et al. Inhibitory effects of seven components of danshen extract on catalytic activity of cytochrome P450 enzyme in human liver microsomes. Drug Metab Dispos 2008 Jul;36(7):1308-14.

(64) Qiu F, Wang G, Zhao Y, Sun H, Mao G, A J, et al. Effect of danshen extract on pharmacokinetics of theophylline in healthy volunteers. Br J Clin Pharmacol 2008 Feb;65(2):270-4.

(65) Qiu F, Wang G, Zhang R, Sun J, Jiang J, Ma Y. Effect of danshen extract on the activity of CYP3A4 in healthy volunteers. Br J Clin Pharmacol 2010 Jun;69(6):656-62.

(66) Wu WW, Yeung JH. Inhibition of warfarin hydroxylation by major tanshinones of Danshen (Salvia miltiorrhiza) in the rat in vitro and in vivo. Phytomedicine 2010 Mar;17(3-4):219-26.

(67) Yu XY, Lin SG, Zhou ZW, Chen X, Liang J, Liu PQ, et al. Role of P-Glycoprotein in the intestinal absorption of tanshinone IIA, a major active ingredient in the root of Salvia miltiorrhiza Bunge. Current Drug Metabolism 2007;8(4):325-40.

(68) Zhang J, Huang M, Guan S, Bi HC, Pan Y, Duan W, et al. A mechanistic study of the intestinal absorption of cryptotanshinone, the major active constituent of Salvia miltiorrhiza. Journal of Pharmacology and Experimental Therapeutics 2006;317(3):1285-94.

(69) Yu XY, Zhou ZW, Lin SG, Chen X, Yu XQ, Liang J, et al. Role of ATP-binding cassette drug transporters in the intestinal absorption of tanshinone IIB, one of the major active diterpenoids from the root of Salvia miltiorrhiza. Xenobiotica 2007 Apr;37(4):375-415.

(70) Hooyenga PA, Witkamp RF, Groen K. Herbal products: Marketing strategies and legislation. International Journal of Green Pharmacy 2009;3(4):270-6.

(71) Azar RR, Badaoui G, Sarkis A, Azar M, Aydanian H, Harb S, et al. Effect of ezetimibe/atorvastatin combination on oxidized low density lipoprotein cholesterol in patients with coronary artery disease or coronary artery disease equivalent. Am J Cardiol 2010 Jul 15;106(2):193-7.

(72) Verberk WJ, Kroon AA, Kessels AG, Lenders JW, Thien T, van Montfrans GA, et al. The optimal scheme of self blood pressure measurement as determined from ambulatory blood pressure recordings. J Hypertens 2006 Aug;24(8):1541-8.

(73) van Tits LJ, de WF, Hak-Lemmers HL, van HP, de GJ, Demacker PN, et al. Effects of alpha-tocopherol on superoxide production and plasma intercellular adhesion molecule-1 and antibodies to oxidized LDL in chronic smokers. Free Radic Biol Med 2001 May 15;30(10):1122-9.

(74) Benzie IF, Strain JJ. The ferric reducing ability of plasma (FRAP) as a measure of "antioxidant power": the FRAP assay. Anal Biochem 1996 Jul 15;239(1):70-6.

(75) Dekker D, Dorresteijn MJ, Pijnenburg M, Heemskerk S, Rasing-Hoogveld A, Burger DM, et al. The bilirubin-increasing drug atazanavir improves endothelial function in patients with type 2 diabetes mellitus. Arterioscler Thromb Vasc Biol 2011 Feb;31(2):458-63.

(76) van Geffen M, Loof A, Lap P, Boezeman J, Laros-van Gorkom BA, Brons P, et al. The Nijmegen Haemostasis Assay: an assay for simultaneous measurement of the thrombin and plasmin potential. Submitted 2011.

(77) Pakala R, Waksman R. Currently available methods for platelet function analysis: advantages and disadvantages. Cardiovasc Revasc Med 2010 Oct 29.

(78) Castaman G, Tosetto A, Goodeve A, Federici AB, Lethagen S, Budde U, et al. The impact of bleeding history, von Willebrand factor and PFA-100((R)) on the diagnosis of type 1 von Willebrand disease: results from the European study MCMDM-1VWD. Br J Haematol 2010 Nov;151(3):245-51.

(79) Pop GA, de Backer TL, de JM, Struijk PC, Moraru L, Chang Z, et al. On-line electrical impedance measurement for monitoring blood viscosity during on-pump heart surgery. Eur Surg Res 2004 Sep;36(5):259-65.

(80) Chen H, Sullivan G, Yue LQ, Katz A, Quon MJ. QUICKI is a useful index of insulin sensitivity in subjects with hypertension. Am J Physiol Endocrinol Metab 2003 Apr;284(4):E804-E812.

(81) Chen H, Sullivan G, Quon MJ. Assessing the predictive accuracy of QUICKI as a surrogate index for insulin sensitivity using a calibration model. Diabetes 2005 Jul;54(7):1914-25.

(82) Liu Y, Li X, Li Y, Wang L, Xue M. Simultaneous determination of danshensu, rosmarinic acid, cryptotanshinone, tanshinone IIA, tanshinone I and dihydrotanshinone I by liquid chromatographic-mass spectrometry and the application to pharmacokinetics in rats. J Pharm Biomed Anal 2010 Nov 2;53(3):698-704.

(83) Han DE, Gao ZD, Zhao D, Wang L, Li N, Li TT, et al. Liquid chromatography mass spectrometry for the determination of salvianolic acid B, a natural compound from the herb Danshen in rat plasma and application to pharmacokinetic study. Biomed Chromatogr 2009 Oct;23(10):1073-8.

(84) Zhang J, Yu H, Sheng Y, Li L, Ye M, Guo D. HPLC determination and pharmacokinetic studies of salvianolic acid B in rat plasma after oral administration of Radix Salviae Miltiorrhizae extract. Biomed Chromatogr 2005 Jan;19(1):15-8.

1. For time schedule and frequency of assessments, see paragraph “Study Design”. [↑](#footnote-ref-1)
