# Supplementary material for: Salvia Miltiorrhiza Root Water-Extract (Danshen) Has No Beneficial Effect on Cardiovascular Risk Factors. A Randomized Double-Blind Cross-Over Trial
Source: PLoS One. 2015 Jul 20;10(7):e0128695. doi: 10.1371/journal.pone.0128695 (PMC4508048; doi:10.1371/journal.pone.0128695)
Supplement: S3 Text — (DOCX) [file pone.0128695.s003.docx]

**Pulse diagnosis**

According to TCM-practitioners, the typical pulse for “blood stasis” is choppy but powerful, which is traditionally explained by the low mobility of blood or by the lack of thin fluid substances within the blood. However, as “blood stasis” usually occurs in combination with other syndromes, there may be a deep, wiry, tense or slow pulse, depending on the concomitant syndrome.

**Tongue diagnosis**

Dark blue or purple dots on the tongue with a purple shading of the tongue body generally is qualified by TCM-practitioners as ‘blood stasis’. Furthermore, when the sublingual veins are dark blue or deep purple, or crooked, then in most cases ‘blood stasis’ is present according to the qualification by TCM-practitioners.
