# Supplementary material for: Salvia Miltiorrhiza Root Water-Extract (Danshen) Has No Beneficial Effect on Cardiovascular Risk Factors. A Randomized Double-Blind Cross-Over Trial
Source: PLoS One. 2015 Jul 20;10(7):e0128695. doi: 10.1371/journal.pone.0128695 (PMC4508048; doi:10.1371/journal.pone.0128695)
Supplement: S4 Text — (DOCX) [file pone.0128695.s004.docx]

**Funding and conflicts of interest**

This study was funded by Cinmar Pharma BV, ‘s Hertogenbosch, the Netherlands, Department of Pharmacology-Toxicology, Radboud university medical centre, Nijmegen, the Netherlands, and the Dutch Association of Acupuncture Medicine (NAAV, Amsterdam, the Netherlands).

Cinmar Pharma had no role in study design, data collection and analysis, decision to publish, or preparation of the manuscript.

AHT is a representative of NAAV and was involved in preparation of the manuscript. He nor NAAV have any financial interest in Danshen.

After preparation of the study, PB left the University and founded a small company (Energeia Biosciences BV, Geertruidenberg, the Netherlands) involved in research and marketing of TCM products. PB reports grants from Cinmar Pharma, during the conduct of the study; In addition, Dr. Breedveld has a patent “Method for identifying modulators of BCRP/ABCG2-mediated ATP release and use of said modulators for treating diseases” pending.

However, this patent is not related to the research in this manuscript, but is related to (plant-derived) immunomodulators based on modulation of BCRP/ABCG2-mediated ATP release, including TCM-derived compounds, such as Danshen-derived compounds.

This does not alter our adherence to PLOS ONE policies on sharing data and materials. All the authors have declared that no competing interests exist.
